# Cost analysis and efficacy of recruitment strategies used in a large pragmatic community-based clinical trial targeting low-income seniors: a comparative descriptive analysis

**DOI:** 10.1186/s13063-019-3652-5

**Published:** 2019-10-07

**Authors:** Sravya Kakumanu, Braden J. Manns, Sophia Tran, Terry Saunders-Smith, Brenda R. Hemmelgarn, Marcello Tonelli, Ross Tsuyuki, Noah Ivers, Danielle Southern, Jeff Bakal, David J. T. Campbell

**Affiliations:** 1 Department of Biological Sciences, Faculty of Science, University of Calgary, Calgary, AB Canada; 2 Department of Medicine, Cumming School of Medicine, University of Calgary, TRW 3E33, 3280 Hospital Dr NW, Calgary, AB T2N 4Z6 Canada; 3 Department of Community Health Sciences, Cumming School of Medicine, University of Calgary, Calgary, AB Canada; 4 Department of Pharmacology, Faculty of Medicine and Dentistry, University of Alberta, Edmonton, AB Canada; 5 Department of Family & Community Medicine, Faculty of Medicine, University of Toronto, Toronto, ON Canada; 6 Department of Family & Community Medicine, Women’s College Hospital, Toronto, ON Canada; 7 Department of Medicine, Faculty of Medicine and Dentistry, University of Alberta, Edmonton, AB Canada; 8 Department of Cardiac Sciences, Cumming School of Medicine, University of Calgary, Calgary, AB Canada

**Keywords:** Randomized controlled trials, Vulnerable populations, Cost-effective, Recruitment strategies, Seniors, Low enrollment

## Abstract

**Objective:**

One of the most challenging parts of running clinical trials is recruiting enough participants. Our objective was to determine which recruitment strategies were effective in reaching specific subgroups.

**Study design and setting:**

We assessed the efficacy and costs of the recruitment strategies used in the Assessing Outcomes of Enhanced Chronic Disease Care Through Patient Education and a Value-based Formulary Study (ACCESS) in Alberta, Canada.

**Results:**

Twenty percent of the study budget ($354,330 CAD) was spent on recruiting 4013 participants, giving an average cost per enrolled of $88 CAD. Pharmacies recruited the most participants (*n* = 1217), at a cost of $128/enrolled. ”Paid media” had the highest cost ($806/enrolled), whereas ”word of mouth” and ”unpaid media” had the lowest (~$3/enrolled). Participants enrolled from ”seniors outreach” had the lowest baseline quality of life and income, while participants from ”word of mouth” had the lowest educational attainment.

**Conclusion:**

The ”health care providers” strategies were especially successful — at a moderate cost per enrolled. The "media" strategies were less effective, short lasting, and more costly. No strategy was singularly effective in recruiting our targeted groups, emphasizing the importance of utilizing a variety of strategies to reach recruitment goals.

**Trial registration:**

ClinicalTrials.gov, NCT02579655. Registered on 19 October 2015.

**Electronic supplementary material:**

The online version of this article (10.1186/s13063-019-3652-5) contains supplementary material, which is available to authorized users.

## Introduction

Randomized controlled trials (RCTs) are widely recognized as the most robust study design for clinical research [1]. One of the greatest challenges in the conduct of clinical trials is participant recruitment [2]. A review of 253 terminated trials found that almost 40% were discontinued prematurely due to difficulties with recruitment [3]. Another study found that up to 50% of trials had to be extended to enroll a sufficient number of participants, and yet only about 30% of clinical trials meet their recruitment targets [4, 5]. As a result of under-recruitment, many studies suffer from a lack of statistical power [6]. This has potential risks: if researchers cannot make their study worthwhile, they waste participants’ time and expose them to unnecessary risks without the promise of improvements in clinical care, which is both unproductive and unethical [6, 7]. Recruitment challenges also result in considerable additional costs to extend study recruitment periods [8], which may deter funding institutions from supporting clinical trials [6].

Numerous authors have attempted to explain why recruitment for clinical trials is so difficult. Some have described problems related to study design [2, 9–13] and recruitment methodology, such as undesirable trial arms (i.e., control groups) and ineffective recruitment strategies for the target audience [12, 14, 15]. Others have noted challenges specifically inherent to the potential participants [2, 6, 11–13] and researchers [2, 12, 13].

Some recent clinical trials have included detailed descriptions about their recruitment process in the published literature. A recent Australian study of older men with diabetes and low testosterone showed successful recruitment through radio and TV advertisements as well as government mail-outs, where all three approaches were relatively cost-effective [16]. Other studies have successfully used health care referrals [16–20], mail-outs [16, 21–23], and community outreach [18, 24]. However, there are still gaps in our knowledge regarding the effectiveness of various recruitment strategies for different target populations [27], the costs associated with them [28], and their success in reaching specific subgroups, which is particularly lacking in the literature. These knowledge gaps are more prominent for researchers attempting to recruit older participants or socially vulnerable participants, as they are often excluded or under-represented in the majority of trials [29].

The objective of this paper was to use our experience with the Assessing Outcomes of Enhanced Chronic Disease Care Through Patient Education and a Value-based Formulary Study (ACCESS trial) to address this knowledge gap and to describe the most effective strategies for researchers seeking to enroll low-income seniors into pragmatic clinical trials. The specific objectives included (1) describing the effectiveness and types of patients enlisted by the various recruitment strategies used, (2) detailing the costs associated with each of these strategies, and (3) determining the cost per enrolled participant for each of these strategies.

## Methods

### Setting

The ACCESS trial is a pragmatic clinical trial in Alberta, Canada, which completed recruitment in August 2018 (ClinicalTrials.gov registration NCT02579655). ACCESS is a factorial (2 × 2) RCT that is evaluating the impact of providing free high-value medications and/or a tailored health education program on patient outcomes and health care costs among seniors at high risk of cardiovascular complications [30]. The free medications intervention involved elimination of patients’ 25% copayments for all medications considered high value in preventing cardiovascular disease (Additional file 1). The personalized education intervention included an online and/or mail-based platform through which participants received tailored health messages. Eligible participants were older people (> 65 years) with low incomes (< $50,000 CAD) who had at least one cardiovascular-related chronic disease (coronary artery disease, congestive heart failure, stroke, diabetes, hypertension, chronic kidney disease, hypercholesterolemia). Randomization was conducted in a blinded fashion using permuted block sizes, and group allocation was in a 1:1:1:1 ratio. Participation involved no study visits, and participants were expected to complete three surveys over the course of the 36-month follow-up period. Most outcomes are being tracked using administrative health data. The trial is ongoing, and follow-up is expected to be complete in September 2021.

The ACCESS trial enrolled 4750 participants from November 2015 to August 2018. The present report is an observational study nested within this RCT, based on data from the first 4013 participants enrolled (November 15, 2015 to May 2, 2018).

Interested participants were asked to call a telephone survey unit, regardless of the recruitment strategy by which they initially heard about the ACCESS trial. Patients were instructed to leave a voicemail, and the survey unit would return their call within 48 h to confirm the participant’s interest and complete an initial eligibility assessment. Participants who qualified for the study and enrolled were asked to complete a baseline survey.

### Data sources

**Fig. 1:**
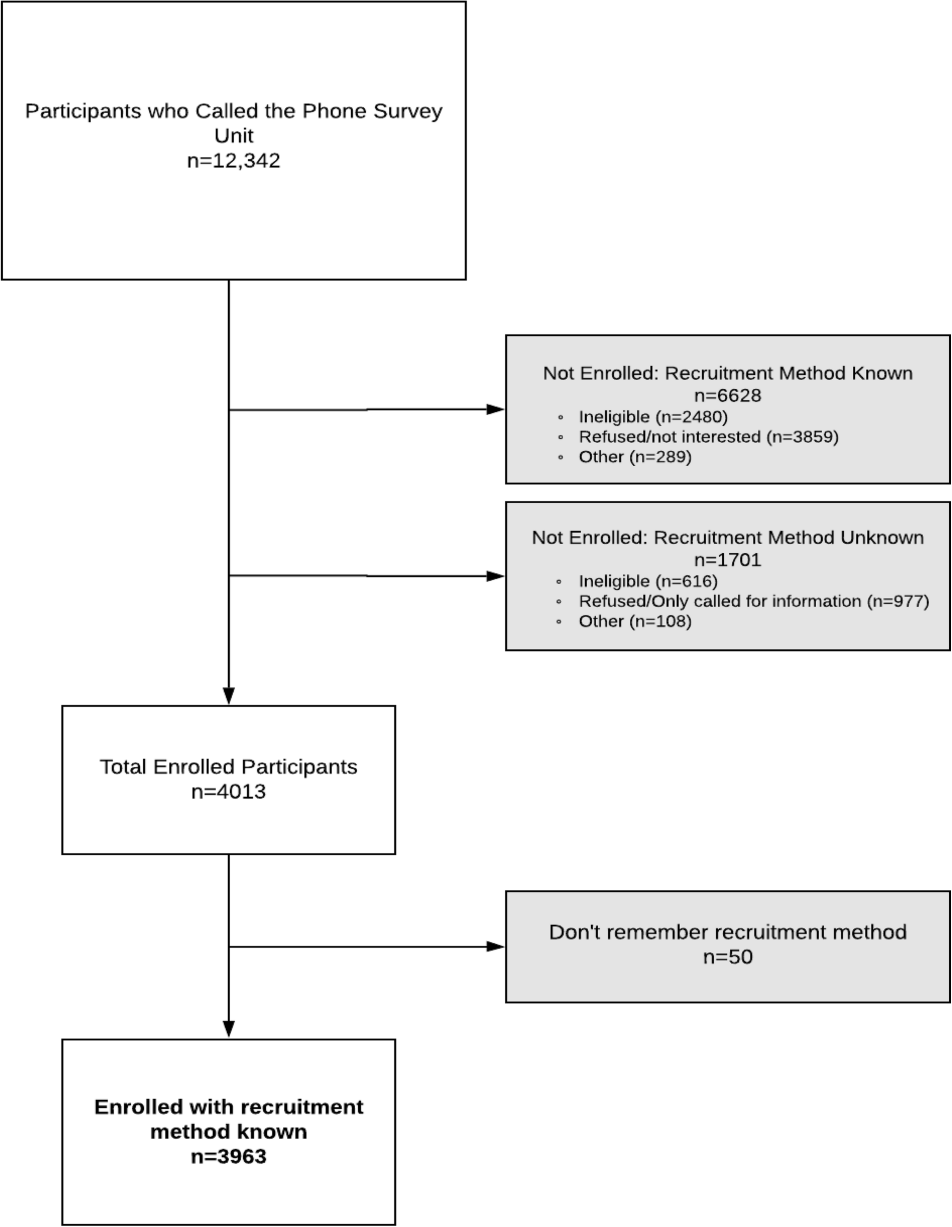
Flow diagram of potential ACCESS trial participants who made contact with the phone survey unit from November 15, 2015 to May 2, 2018

Data for this study was collected by self-report from ACCESS trial participants at the time of their initial contact with study staff. Recruitment strategy and basic demographic details were collected during their initial telephone assessment with the survey unit. Missing data was present for individuals who called for information but chose not to be screened for eligibility and for those deemed to be ineligible early in the conversation and who declined further questions (Fig. 1). Detailed demographic information was extracted from the surveys administered to participants at their baseline assessment. In order to enroll in the ACCESS trial, baseline questionnaires (Additional files 2 and 3) must be complete; therefore, no missing data was present for those who enrolled in the trial. Cost data was extracted from a detailed review of study expenses and human resource utilization (Additional file 4).

### Variables of interest

#### Types of recruitment strategies

Throughout the 30-month enrollment period, a variety of recruitment strategies were used to identify eligible participants for the ACCESS trial, which we have classified into five overarching strategies and 14 substrategies (Table 1). Participants who called the survey unit were asked an open-ended question to determine how they learned about the study. Responses to this question were allocated to one of the 14 substrategies. Participants who did not remember how they heard about the study were placed in the “Don’t Know” category.

**Table 1:** Recruitment strategies used in the ACCESS trial

1. Patient contact by health care providers	
a. Pharmacies: Several large Canadian pharmacy chains, along with hundreds of independent pharmacies across Alberta, were approached by the ACCESS trial team to be a part of the recruitment process. They were asked to display posters and/or hand out brochures to patients. Pharmacists had considerable autonomy to decide how to recruit patients, with some only having posters displayed, others handing out brochures, and others directly targeting individuals they felt would be appropriate for the study. Regardless, pharmacists did not enroll patients directly but simply provided the number for the study survey unit	
b. Health professionals: Throughout the study, we distributed posters and brochures to specialist and family physician offices as well as hospitals throughout Alberta. This category included participants who saw the posters and/or brochures at these locations and participants who were told about the ACCESS trial by medical or allied health professionals (physicians, nurses, and dieticians) other than pharmacists	
2. Paper mail	
a. Census-based Canada Post mail-out: Brochures were mass mailed to targeted communities identified by Canada Post as having a preponderance of residents who were over the age of 65 and had lower incomes, based on census data. A total of 122,000 brochures were sent out in three separate mailing cycles	
b. Coronary angiogram registry: All consenting Albertans who undergo cardiac catheterization for diagnostic or therapeutic purposes are entered into a patient registry called the Alberta Provincial Project for Outcome Assessment in Coronary Heart Disease (APPROACH) [21]. Those who are found to have coronary artery disease are asked to consent to being contacted to hear about future clinical studies. We used the list of those consenting individuals to contact those with demographics suggesting potential eligibility for ACCESS. These individuals were directly mailed a letter about ACCESS. Everyone on the mail-out list was then called by our survey unit to confirm interest and eligibility. A total of 4780 individuals received letters and phone calls over 11 months	
c. Targeted mail-out after hospital discharge: Patients who were discharged from an Alberta Health Services (AHS) facility (the single health care provider in Alberta) who met study criteria (based on age and previously known health conditions) were contacted by mail by AHS, inviting them to call the survey recruitment line if they were interested in participating. A total of 50,042 letters were sent over 11 months	
3. Media	
a. Paid radio advertisements: Three local radio stations in Calgary and nearby areas were selected on the basis of having a large audience with the correct target demographic to play 30-s commercials up to 42 times a week for 2 weeks. The radio commercial included instructions on how to enroll and a brief summary of the study, highlighting that eligible individuals would have a 50% chance to receive free medications	
b. Facebook: A paid Facebook ad was designed to recruit participants (disseminated to Alberta seniors and a separate Facebook ad to younger individuals—noting the potential relevance to their parents and grandparents) and was displayed on the pages of targeted groups for a few months	
c. Hospital programming channel: A professionally produced 90-s TV commercial aired on televisions in select physicians’ offices, community laboratories, and some AHS facilities throughout Alberta for 6 months in 2016/2017	
d. Transit advertisements: Advertisements were placed at transit stops and stations as well as inside the trains and buses in Calgary, Edmonton, Medicine Hat, and Lethbridge for 11 months	
e. Paid print media: Included anyone recruited from paid print advertisements. This included advertisements placed in general and senior-specific newspapers and newsletters throughout the province	
f. Unpaid media: Included free social media advertising from personal, university, and charitable organizational accounts on Twitter and Facebook. Press releases were sent from the University of Calgary in March 2016 and from AHS in September 2016. A variety of coverage arose from this—predominantly radio stories and interviews. Two news stories featuring the ACCESS trial were broadcast on local TV stations during the evening local news. Various other free stories and articles in papers and newsletters across Alberta arose from a variety of other contacts. Anyone who claimed to see any print, online, or aired media within 3 weeks of the release dates was categorized under “unpaid media”	
4. Seniors outreach	
a. Seniors’ homes: The ACCESS team traveled to and contacted various seniors’ homes and apartments to give presentations and drop off brochures and/or posters. Many rural locations were contacted by phone, and if managers were interested in recruiting, posters and brochures were sent directly to the seniors’ homes. Some seniors' homes posted the materials in common areas, while others put brochures directly in mailboxes or under residents’ doors	
b. Seniors aid resources: Seniors aid resources included social workers, help centers, food banks, and health care or social care service coordinators. Advertising to these places/people consisted of word of mouth and the distribution of brochures to institutions and providers. Any presentations or booths set up by the ACCESS team at events, fairs, or centers were also included in this category	
5. Word of mouth: This strategy was established as the study became more well known. Many study participants told their family members and/or friends about the study and gave them the enrollment phone number. As this strategy became more successful, we further encouraged it by periodically sending enrolled participants recruitment brochures to distribute to those who might be eligible and interested	

#### Classification of participants

We classified potential participants who initially contacted the survey unit for eligibility assessment into two major categories: (1) those who eventually enrolled and were randomized, and (2) those who did not enroll for a variety of reasons. This latter group was further classified as either ”ineligible”, ”refused”, or ”other”. Furthermore, all potential participants who provided the recruitment substrategy by which they heard about the study were categorized into the appropriate overarching recruitment strategy.

For all enrolled participants, we extracted demographic information from the eligibility surveys, including urban versus rural residence, first language, income category, educational attainment, age category, prescribed medications, and quality of life. This was done to determine the effectiveness of the various recruitment strategies at targeting specific populations.

#### Recruitment costs

We also estimated detailed costs associated with each of the 14 strategies. The total costs included both supplies and services costs, as well as human resources costs. Supplies and services costs included expenses for materials (brochures and posters), travel, advertising fees, postage fees, and consulting fees. All promotional content was developed by the study team and was approved by the local ethics boards before distribution. Focus groups conducted later in the study provided some feedback on the promotional content [25]. Human resources costs included the time required by the ACCESS team (research assistants and research coordinator) to start, organize, and sustain the strategy, and, in the case where the participants were actively called by the survey unit (coronary angiogram registry), the time required by the survey unit to call participants (Additional file 4). The human resources costs did not include the time of study investigators or co-investigators who oversaw the recruitment effort.

### Data analysis

#### Efficacy of paper mail strategies

The mail-based strategies provided a unique opportunity to calculate a denominator (i.e., knowing how many people were initially contacted by each strategy). Therefore, we determined how successful each of these strategies were at getting recipients to actually call and enroll in the study. We calculated two measures of effectiveness for each strategy: (1) the proportion of initial contacts who received mail who ended up enrolling in the study (proportion of initial contacts), and (2) the proportion of those who actually called who completed enrollment (proportion of callers). The first percentage was calculated by dividing the number of enrolled by the total number of individuals who were sent mail, and the second percentage was calculated by dividing the number of enrolled by the number of calls received by the survey unit.

#### Demographic analysis

The sociodemographic characteristics of the participants enrolled were compared across recruitment strategies with the goal of determining whether the strategies differed in their ability to reach different types of patients. In particular, we were interested in between-strategy differences in effectiveness of recruiting individuals from typically under-represented and difficult-to-recruit subgroups.

For each recruitment strategy, the proportion of its enrolled participants from each demographic category was calculated, as well as corresponding 95% confidence intervals (CIs) using the binomial distribution. A quality of life measurement was also included as a demographic characteristic of the enrolled participants. Quality of life was calculated using the EuroQol five-dimension, five-level (EQ-5D-5 L) scoring system, a measure of overall well-being that gives a score from 0 to 1, with 1 representing perfect health.

#### Timeline analysis

We were interested in whether media recruitment strategies resulted in sustained interest in the study. We therefore plotted media recruitment by the week of study and superimposed the various media recruitment strategies.

#### Cost analysis

Finally, by using the estimated costs incurred by each strategy, we were able to calculate a strategy-specific cost per enrolled participant. This was calculated by dividing the total cost of the strategy by the number of participants enrolled by the strategy.

## Results

### Study cohort

From November 2015 until May 2018 a total of 12,342 people called the survey unit (Fig. 1). Of these potential participants, 4013 were randomized, of whom 50 were unable to recall how they heard about the study. Of the 8329 potential participants who did not enroll, 1701 did not specify how they heard about the study either because they were disqualified too early during the phone survey to answer the question or they had just called for information, not intending to enroll.

### Analysis of recruitment strategies

#### Demographic analysis

Given that ACCESS was attempting to enroll individuals who were less likely to be on optimal medical therapies, the demographics that were most important to target in this study were older seniors with lower incomes, those not on indicated medications (statins, angiotensin-converting enzyme [ACE] inhibitors/angiotensin receptor blockers [ARBs]) at baseline, those with lower levels of educational attainment, and those with a lower quality of life. Nearly 1/3 (29%) of participants enrolled through the coronary angiogram registry were above the age of 80, making it the strategy with the highest proportion of older seniors (Table 2). Participants from seniors outreach were the most likely to be in the lowest income bracket (74% had an income < $30,000 CAD). Pharmacies recruited the most people with less than a high school education (*n* = 379), but word of mouth, although recruiting fewer than half as many participants, still a slightly higher proportion of its enrolled participants with less than a high school education at 35% (*n* = 166). Only 8% of all participants were not on either class of indicated medication; health professionals and seniors outreach had the highest proportion (12%) of their enrolled participants meeting this criteria. Finally, the utility scores showed that participants from seniors outreach had the lowest quality of life with an average EQ-5D score of 0.617, while participants recruited from the coronary angiogram registry had the highest quality of life with an average score of 0.686.

**Table 2:** Participant demographics, overall and by recruitment strategy

Demographic	TOTAL *N* (% of total enrolled, 95% CI)	Pharmacies *N* (% of total enrolled by the strategy, 95% CI)	Health professionals *N* (% of total enrolled by the strategy, 95% CI)	Canada Post mail-out *N* (% of total enrolled by the strategy, 95% CI)	Coronary angiogram registry *N* (% of total enrolled by the strategy, 95% CI)	Contact after hospital discharge *N* (% of total enrolled by the strategy, 95% CI)	Media *N* (% of total enrolled by the strategy, 95% CI)	Seniors outreach *N* (% of total enrolled by the strategy, 95% CI)	Word of mouth *N* (% of total enrolled by the strategy, 95% CI)
Total enrolled by the strategy	4013	1217	310	198	630	530	350	252	476
Residing location^1^									
Rural	1350 (34%, 32–35)	411 (34%, 31–37)	81 (26%, 21–31)	97 (49%, 42–56)	262 (42%, 38–46)	192 (36%, 32–41)	98 (28%, 23–33)	60 (24%, 19–30)	140 (29%, 25–34)
English understanding									
Does not understand English	466 (12%, 11–13)	269 (22%, 20–25)	33 (11%, 7.4–15)	12 (6%, 3.2–10)	8 (1.3%, 0.64–2.5)	19 (4%, 2.2–5.5)	24 (7%, 4.4–10)	18 (7%, 4.3–11)	78 (16%, 13–20)
Annual household income									
< $15,000	418 (10%, 9.5–11)	186 (15%, 13–17)	44 (14%, 11–19)	9 (5%, 2.1–8.5)	27 (4%, 2.8–6.2)	30 (6%, 3.9–8.0)	16 (5%, 2.6–7.3)	28 (11%, 7.5–16)	69 (14%, 12–18)
$15,000–$29,999	1873 (47%, 45–48)	577 (47%, 45–50)	159 (51%, 46–57)	83 (42%, 35–49)	238 (38%, 34–42)	194 (37%, 33–41)	199 (57%, 52–62)	160 (63%, 57–69)	243 (51%, 47–56)
≥ $30,000	1722 (43%, 41–44)	454 (37%, 35–40)	107 (35%, 29–40)	106 (54%, 46–61)	365 (58%, 54–62)	306 (58%, 53–62)	135 (39%, 33–44)	64 (25%, 20–31)	164 (34%, 30–39)
Education									
< High school	1041 (26%, 25–27)	379 (31%, 29–34)	65 (21%, 17–26)	57 (29%, 23–36)	138 (22%, 19–25)	103 (19%, 16–23)	61 (17%, 14–22)	60 (24%, 19–30)	166 (35%, 31–39)
High school	1140 (28%, 27–30)	351 (29%, 26–32)	87 (28%, 23–33)	56 (28%, 22–35)	187 (30%, 26–33)	144 (27%, 23–31)	98 (28%, 23–33)	68 (27%, 22–33)	135 (28%, 24–33)
Post-secondary	1832 (46%, 44–47)	487 (40%, 37–43)	158 (51%, 45–57)	85 (43%, 36–50)	305 (48%, 44–52)	283 (53%, 49–58)	191 (55%, 49–60)	124 (49%, 43–56)	175 (37%, 32–41)
Age category									
65–70 years	1364 (34%, 33–36)	555 (46%, 43–49)	152 (49%, 43–55)	55 (28%, 22–35)	75 (12%, 9.5–15)	106 (20%, 17–24)	137 (39%, 34–45)	105 (42%, 36–48)	166 (35%, 31–39)
71–80 years	1922 (48%, 46–50)	503 (41%, 39–44)	119 (38%, 33–44)	106 (54%, 46–61)	372 (59%, 55–63)	316 (60%, 55–64)	159 (45%, 40–51)	102 (40%, 34–47)	218 (46%, 41–50)
> 80 years	727 (18%, 17–19)	159 (13%, 11–15)	39 (13%, 9.1–17)	37 (19%, 14–25)	183 (29%, 26–33)	108 (20%, 17–24)	54 (15%, 12–20)	45 (18%, 13–23)	92 (19%, 16–23)
Medications^2^									
Not on either	328 (8%, 7.3–9.1)	80 (7%, 5.2–8.1)	36 (12%, 8.3–16)	14 (7%, 3.9–12)	30 (5%, 3.2–6.7)	55 (10%, 7.9–13)	33 (9%, 6.6–13)	29 (12%, 7.8–16)	46 (10%, 7.2–13)
On only one	1308 (33%, 31–34)	385 (32%, 29–34)	92 (30%, 25–35)	82 (41%, 34–49)	176 (28%, 25–32)	186 (35%, 31–39)	132 (38%, 33–43)	78 (31%, 25–37)	161 (34%, 30–38)
On both	2377 (59%, 58–61)	752 (62%, 59–65)	182 (59%, 53–64)	102 (52%, 44–59)	424 (67%, 64–71)	289 (55%, 50–59)	185 (53%, 47–58)	145 (58%, 51–64)	269 (57%, 52–61)
Gender									
Women	1868 (47%, 45–48)	601 (49%, 47–52)	147 (47%, 42–53)	86 (43%, 36–51)	188 (30%, 26–34)	220 (42%, 37–46)	178 (51%, 45–56)	143 (57%, 50–63)	288 (61%, 56–65)
Quality of life scores^3^ Mean (95% CI*)*	0.653 (0.649–0.657)	0.653 (0.645–0.661)	0.637 (0.621–0.653)	0.658 (0.639–0.677)	0.686 (0.676–0.696)	0.635 (0.635–0.636)	0.658 (0.645–0.671)	0.617 (0.599–0.636)	0.649 (0.649–0.650)

Strategies with individual substrategies that had large enough sample sizes to be analyzed separately or were successful at targeting different demographics were differentiated

^1^Urban areas were classified as having a population ≥ 25,000

^2^Two categories of medications were important to this study: statins and ACEs/ARBs. Participants were classified as being prescribed or not prescribed these groups of medications

^3^Quality of life scores were calculated using the EQ-5D-5 L scoring system

#### Recruitment success

The largest proportion of our 4013 participants was enrolled from health care providers (*n* = 1527; 38%), with the largest group coming from pharmacies (*n* = 1217; 30%) (Table 2). The mail-based strategies recruited 1358 participants (34% of the total), while paid media only recruited 85 individuals (2% of all participants). Each of the individual strategies under paid media recruited fewer participants than any other strategy used in the study (Table 3).

**Table 3:** Summary of participants enrolled and cost breakdown, by recruitment strategy

Recruitment strategy	Number of enrolled *N* (% of total enrolled, 95% CI)	Supplies and services cost ($CAD)	Human resources cost ($CAD)	Total cost ($CAD)	Cost per enrolled $CAD/participant
Health care	1527 (38%, 37–40)	66,690	91,910	158,600	104
Pharmacies	1217 (30%, 29–32)	63,500	91,480 ^a^	154,980	128
Health professionals	310 (7.7%, 6.9–8.6)	3190	430 ^a^	3620	12
Paper mail	1358 (34%, 32–35)	90,770	15,370	106,140	78
Canada Post mail-out (*n* = 122,000)	198 (4.9%, 4.3–5.7)	39,400	300 ^a^	39,700	201
Coronary angiogram registry (*n* = 4780)	630 (16%, 15–17)	4780	12,670^b^	17,450	28
Contact after hospital discharge (*n* = 50,042)	530 (13%, 12–14)	46,590	2400^b^	48,990	92
Media	350 (8.7%, 7.9–9.6)	66,610	2940	69,550	199
Paid media	85 (2.1%, 1.7–2.6)	66,610	2040	68,650	808
Paid radio	13 (0.32%, 0.19–0.55)	11,850	120	11,970	921
Facebook	2 (0.050%, 0.014–0.018)	10,200	300^a^	10,500	5250
Hospital programming channel	8 (0.20%, 0.10–0.39)	10,220	600^a^	10,820	1353
Transit advertising	26 (0.65%, 0.44–0.95)	23,040	120	23,160	891
Print media	36 (0.90%, 0.65–1.2)	11,300	900^a^	12,200	339
Unpaid media	265 (6.6%, 5.9–7.4)	0	900^a^	900	3
Seniors outreach	252 (6.3%, 5.6–7.1)	12,380	4260	16,640	66
Senior’s homes/apartments	74 (1.8%, 1.5–2.3)	5690	4100^a^	9790	132
Senior’s aid resources	178 (4.4%, 3.9–5.2)	6690	160^a^	6850	38
Word of mouth	476 (12%, 11–13)	2200	1200	3400	7
TOTAL	4013	238,650	115,680	354,330	88

^a^Cost calculated using research assistant salary at approximately $30 CAD/h

^b^Cost calculated using research coordinator salary at approximately $60CAD/h

**Fig. 2:**
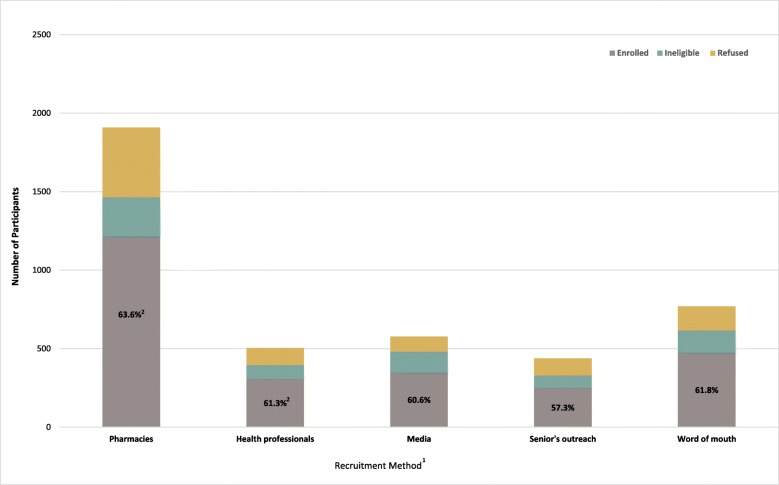
Proportion of callers from each method who enrolled. ^1^This data for the mail methods is excluded from this figure and presented separately (Table 4) due to its much larger sample size and known denominator. ^2^The individual strategies under Health Care Providers are analyzed separately due to their significant differences in sample size and the success and expenditure of pharmacies alone as a strategy

Despite this broad variation in success in prompting individuals to call the survey unit, the recruitment strategies generally seemed to enroll a similar proportion of initial callers (~ 60%) (Fig. 2). However, this value was only calculated using the potential participants who provided their recruitment strategy; 1701 callers did not provide this information (Fig. 1.) The exception to this generalization was the mail strategies, where there was considerably more variation (Table 4).

**Table 4:** Effectiveness of mail strategies

Mail-out strategy	Initial Contacts (total mailed)	Number of calls into VOXCO *N* (% callers out of total mailed)	Contacts who ended up enrolling in the study		
			*N*	% enrolled out of total mailed	% enrolled from total number of calls
*All mail strategies*	*176,822*	*6384 (3.61%)*	*1358*	*0.768%*	*21.3%*
Canada Post mail-out	122,000	300 (0.246%)	198	0.162%	66.0%
Coronary angiogram registry	4780	4780 (100%)^a^	630	13.2%	13.2%
Contact after hospital discharge	50,042	1304 (2.61%)	530	1.06%	40.6%

^a^This strategy was unique in that we actively called all contacts, rather than simply providing them with the survey unit phone number

#### Mail strategies

Of the 176,822 people who were initially contacted by mail, only 3.61% (*n* = 6384) contacted the telephone survey unit (Table 4). Furthermore, only 21.3% (*n* = 1358) of these callers were eligible and consented to enrollment, which meant only 0.8% (1358/176,822) of all people contacted by mail actually enrolled in the study. Regarding the specific mail strategies: the coronary angiogram registry had 100% (*n* = 4780) of contacts complete the phone survey (due to the fact that we actively called them). However, with only 630 of the potential participants enrolling, it successfully enrolled the lowest percentage of those who were spoken to on the phone (13.2%) compared to the other mail-based strategies. On the other hand, the Canada Post mail-out had the lowest proportion of contacts who called in (0.25%, *n* = 300), but those who called in were much more likely to enroll, with a proportion similar to the non-mail strategies (66.0%, *n* = 198). The contact after hospital discharge strategy only had 2.61% (*n* = 1304) of its initial contacts call the phone survey unit, and with 40.6% enrolling (*n* = 530), it had a smaller proportion of its initially contacted participants enroll, compared to other strategies.

#### Recruitment timeline

**Fig. 3:**
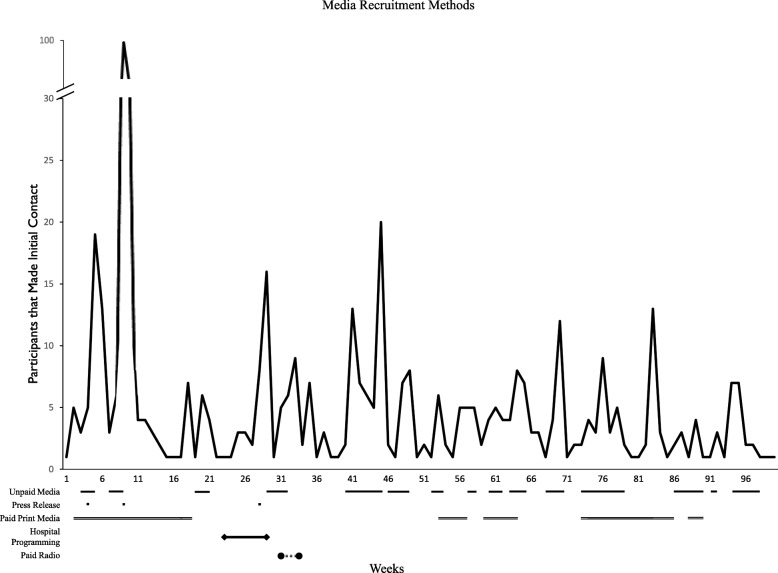
Number of people who called the survey unit during each week of the study’s recruitment period with duration of media strategy implementation

Various recruitment strategies caused discrete and noticeable increases in recruitment. Particularly, the press releases, and some of the unpaid media efforts, resulted in a measurable uptick in recruitment. Notably, none of the strategies seemed to cause any degree of sustained recruitment, as each increase only lasted for 1–2 weeks. The first press release was considerably more impactful than the second. Many of the smaller spikes in the later weeks of the study did not coincide with our paid media strategies and could only be attributed to unpaid media, or word of mouth (Fig. 3).

### Costs of recruitment strategies

The total cost of recruitment was $354,330 CAD (Table 3). Nearly half of this sum was spent on health care provider recruitment ($158,600), which was the most successful strategy. As per the recruitment plan, most of the human resources were spent on pharmacies rather than other health professionals because they were located in the community, they tended to be more inclined to become involved with research, and people were likely to contact their pharmacist often. The study team spent most of their time informing pharmacies about the ACCESS trial and continuously sent out posters and pamphlets to enable them to advertise for us. Involvement of other health providers in recruitment was done on an ad hoc basis, when the team was contacted by providers looking to get involved, and therefore required much less employee time. The least costly recruitment strategies were word of mouth ($3400 CAD) and unpaid media ($900 CAD), which both recruited small numbers of participants. The cost per enrolled participant varied greatly between and within the overarching strategies, with an overall average cost of recruitment of $88 CAD per enrolled participant (Table 3). Of the recruitment strategies, paid media had the highest cost per enrolled at $808 CAD/participant, which included paid radio ($921 CAD/participant), Facebook ($5250 CAD/participant), hospital programming channel ($1353 CAD/participant), transit advertising ($891 CAD/participant), and print media ($339 CAD/participant). This is in contrast to unpaid media, which had the lowest cost per enrolled of all 14 substrategies at $3 CAD/participant. Despite the high total cost of the pharmacy substrategy, its cost per enrolled was only slightly above the average at $128 CAD/participant.

## Discussion

Recruitment for ACCESS was time-consuming and costly, but ultimately successful. We used 14 substrategies to recruit the first 4013 participants into the study, at a cost of $354,330 CAD, which was approximately 20% of the overall study operating expenditures during this period. Despite eventual success, there was a lack of adequate planning and budgeting at the beginning of the study to successfully reach the target number of participants. Initial planning set out 12 months for recruitment, with only $20,000 CAD set aside as a dedicated recruitment budget to contact pharmacies, which was to be the sole recruitment strategy. As recruitment continued, the timeline had to be extended and considerably more resources had to be put towards recruitment—all of the other strategies developed once it became clear that the initial plans for recruitment were going to be inadequate. It is important for researchers to understand how much of the study budget may need to be spent on recruitment, and to plan their funding requests accordingly. No single strategy appeared to succeed in recruiting typically under-represented groups; rather, the strategies differed in their ability to recruit various types of people. This suggests that the use of multiple and varied recruitment strategies is important to increase the likelihood of achieving sociodemographic diversity [2, 12, 26]. The average cost per enrolled participant was $88 CAD; however, the costs involved with each strategy used in the ACCESS trial varied considerably. It is important to recognize how the availability of resources can restrict recruitment efforts, thus influencing what is seen as a successful recruitment strategy. As found in the ACCESS trial, each strategy differs in its cost, ability to recruit participants (including specific types), and timeliness.

Under-recruitment and the high costs of recruiting for clinical trials have been well documented in the literature. Many authors have provided insight on how to change study designs to help researchers reach recruitment goals within their desired budget and time frame [9]. Building upon this work, there were knowledge gaps our study was able to address, most importantly, how to recruit populations which are typically underrepresented in clinical research. Most studies that have analyzed the factors that impact recruitment (including the studies included in Treweek’s meta-analysis [9]) were enrolling younger and healthier participants and not actively targeting low-income seniors with chronic diseases (at high risk of hospitalization). Another important difference is that our study concentrated on understanding the effectiveness of specific recruitment strategies and their associated costs, whereas Treweek’s work had examined other aspects of the study design that can affect recruitment success (i.e., the consent process, the initial information given to potential participants, incentives, trial conduct, etc.) [9]. The study published by Bracken et al. [16] provides a cost analysis and detailed explanations of many commonly used and successful strategies such as radio and TV advertisements, health care referrals, mail-outs, and community outreach which were also used in the ACCESS trial; however, our study targeted a different participant base and also provides a demographic analysis of the subtypes of participants recruited by each strategy. The availability of both of these studies provides future researchers with greater insight on the types of participants to expect from various recruitment strategies, depending on their target demographic. By thoroughly analyzing each individual strategy, our paper provides researchers different perspectives on what could be considered a successful recruitment strategy, allowing them to determine which might be most suitable for their studies.

A common barrier to the recruitment of older participants is lack of trust in the researchers and recruiters [11–13, 26]. This implies that recruitment may be more successful if participants hear about studies from trusted individuals, such as their physicians or pharmacists [2, 27], and it might explain why health care providers enrolled the most participants in our study. Similarly, personal referrals have been shown to be quite successful at persuading people to enroll [2, 11–13, 26, 28, 32, 33]; which we also noted with the success of the ”word of mouth” strategy. Mail-based recruitment strategies can be challenging to implement. Suggestions for improving the success of mail-based recruitment strategies include personalizing the letters as much as possible using a pre-specified list that is more likely to include only individuals who are most likely to be eligible and interested [26]. These factors might explain why two of our mail strategies (coronary angiogram registry and contact after hospital discharge) were considerably more successful than the more generic Canada Post mail-out [2, 11, 26, 34]. However, it is important to note that even though the coronary angiogram registry had a high human resources cost, the active approach of calling participants directly made this strategy much more successful than the other mail strategies, which were more passive. Community-based approaches and cultural adaptation are known to enhance recruitment efforts [11]—which we saw in the moderate success of our outreach strategies. In terms of the ratio of men and women enrolled, each strategy had a relatively equal number of men and women recruited with the exception ”word of mouth” and the coronary angiogram registry. ”Word of mouth” and ”seniors outreach” recruited a higher number of females, potentially because females have been observed to be slightly more social than males [35]. The coronary angiogram registry recruited a higher proportion of males, most likely due to the fact that on average males have cardiovascular events earlier than females [36], making them more likely to be on the coronary angiogram registry.

As we observed, social media is typically thought to be more effective at reaching younger populations than low-income seniors who may not be as technologically inclined [13, 15, 27]. Previous studies have found the total costs of media recruitment to be relatively low [32], which was true for ACCESS as well. However, we had very few participants recruited by paid media, making its cost per enrolled participant quite high. In contrast to paid media, we found that unpaid media (such as coverage stemming from press releases) had the lowest cost per enrolled out of all strategies. This emphasizes the importance of using free advertising and building trust with local communities to help with the promotion of study recruitment [14]. Media coverage seemed to generate immediate interest in the study that was not sustained over time. It is quite clear that the first major press release in March 2016, which garnered much interest in unpaid print and online media, generated a clear spike in initial contacts.

Our analysis has some limitations that merit discussion. First, due to the contextual nature of this work, the findings of this study may only be generalizable to researchers studying a similar population (low-income seniors with chronic diseases) who are offering the possibility of a tangible benefit to study participants (i.e., free medications). Furthermore, the specific costs, and particularly those related to human resources, are highly contextual. Other researchers should anticipate that the exact costs of specific strategies will vary within their own locations, but the relative costs should be generalizable to all settings of similar studies. Second, we do not have detailed information on why individuals declined to participate, which could be useful in the future to modify recruitment approaches. Third, we were unable to account for the impact that media promotion may have had on other recruitment strategies. It is possible that a number of health care providers, senior outreach workers, and friends and family members who referred participants to our study initially heard about it from media sources. Similarly, participants might have heard about the study from multiple sources, but only called the study after the most recent contact. Therefore, the reach of some strategies may have been underestimated. Finally, this quantitative analysis does not offer any insights into why some strategies were more effective or how success was achieved. This would necessitate a qualitative inquiry, which may be informative to researchers - such an analysis has been conducted by our team separately [25].

## Conclusion

In summary, we found that different recruitment strategies were better at targeting certain demographic groups and varied in their overall effectiveness. Overall, recruitment through personal referrals from health care providers was the most expensive but enrolled the most participants. However, strategies such as unpaid media and word of mouth—while enrolling fewer participants—were associated with the lowest cost per enrolled, highlighting the importance of community outreach methods in recruiting populations that are typically under represented in clinical trials. The experience of the ACCESS trial emphasizes the importance of understanding the target demographic of a clinical study and determining the most appropriate strategies to effectively recruit those desired individuals.

## Additional files

Additional file 1: List of covered medications. (DOCX 34 kb)

Additional file 2: Telephone Baseline Questionnaire. (DOCX 66 kb)

Additional file 3: Paper Baseline Survey. (DOCX 612 kb)

Additional file 4: Summary of human resources costs. (DOCX 20 kb)

## Data Availability

The datasets used and/or analyzed during the current study may be made available from the corresponding author on reasonable request.

## Abbreviations

ACCESS: Assessing Outcomes of Enhanced Chronic Disease Care Through Patient Education and a Value-based Formulary Study
ACE: Angiotensin-converting enzyme
ARB: Angiotensin receptor blocker
RCT: Randomized controlled trial
