# Supplementary material for: Cost analysis and efficacy of recruitment strategies used in a large pragmatic community-based clinical trial targeting low-income seniors: a comparative descriptive analysis
Source: Trials. 2019 Oct 7;20:577. doi: 10.1186/s13063-019-3652-5 (PMC6781395; doi:10.1186/s13063-019-3652-5)
Supplement: Supplementary file 1 — List of covered medications. (DOCX 34 kb) [file 13063_2019_3652_MOESM1_ESM.docx]

**Antiarrhythmics**

Disopyramide *(Rythmodan*)

Procainamide *(Procan)*

Mexilentine *(Mexilentine)*

Flecainide (Tambocor)

Propafenone *(Propafenone/Rythmol)*

Amiodarone *(Amiodarone/Cordarone)*

Digoxin *(Toloxin)*

**Nitrates and Nitrites**

Isosorbide Dinitrate (*Cedocard-SR)*

Isosorbide-5-Mononitrate *(Imdur)*

Nitroglycerin *(Nitrostat/Nitro/Nitrolingual/Nitro-dur/Trinipatch/Minitran/Nitrol)*

**Statins**

Atorvastatin *(Lipitor)*

Rosuvastatin *(Crestor)*

Simvastatin *(Zocor)*

Pravastatin *(Pravachol)*

Fluvastatin *(Lescol)*

Lovastatin *(Mevacor)*

**Non-statin Cholesterol Lowering Drugs**

Cholestyramine *(Olestyr)*

Colesevelam *(Lodalis)*

Colestipol *(Colestid)*

Bezafibrate *(Bezalip)*

Fenofibrate *(Feno-micro/Feno-Super/Lipidil Supra)*

Gemfibrozil (Lopid)

Ezetimibe *(Ezetrol)*

Evolulocumab (*Repatha*)

**Beta Blockers**

Acebutalol *(Sectral)*

Atenolol *(Tenormin/Tenoretic/Atenidone/*

*Atenolthalidone)*

Bisoprolol (Zebeta)

Carvedilol (Coreg, Coreg CR)

Labetalol *(Trandate)*

Metoprolol *(Lopresor)*

Propranolol (Inderal)

Sotalol (Betapace)

Nadolol *(Nadol)*

**ACE-inhibitors**

Benazepril *(Lotensin)*

Cilazepril *(Inhibace/Inhibace Plus)*

Enalapril *(Vasotec/ Vaseretic)*

Perindopril *(Coversyl/Coversyl Plus)*

Captopril *(Capoten)*

Fosinopril  *(Monopril)*

Lisinopril *(Zestril/Prinivil/Zestoretic)*

Ramipril *(Altace)*

Quinapril *(Accupril/Accuretic)*

Trandolapril *(Mavik)*

**Angiotensin Receptor Blockers**

Candesartan *(Atacand/Atacand Plus)*

Eprosartan *(Teveten/Teveten Plus)*

Irbesartan *(Avapro / Avalide)*

Losartan *(Cozaar / Hyzaar)*

Telmisartan *(Micardis/Twynsta/ Micardis Plus)*

Valsartan *(Diovan)*

Olmesartan *(Olmetec/Olmetec Plus)* Entresto (*Sacubitril/Valsartan*)

**Calcium Channel Blockers**

Nifedipine *(Adalat XL)*

Amlodipine *(Norvasc)*

Felodipine *(Plendil)*

Diltiazem *(Diltiaz/Cardizem / Tiazac)*

Verapamil *(Verap/Isoptin SR)*

**Diuretics**

Hydrochlorothiazide *(Hydrazide/Hydro)*

Furosemide *(Lasix)*

Spironolactone *(Aldactone / Aldactazide)*

Indapamide *(Lozide)*

Metolazone *(Zaroxolyn)*

Chlorthalidone (Thalitone)

Amiloride *(Midamor/ Novamilor /Amilizide)*

Triamterene *(Triazide*)

Ethacrynic Acid *(Edecrin)*

**Anti-platelet Agents**

Clopidogrel *(Plavix)*

ASA*-Dipyridamole (Aggrenox)*

Ticagrelor *(Brilinta)**

**Anti-diabetes Medications**

Metformin *(Glucophage)*

Glipizide (Glucotrol)

Gliclazide *(Diamicron)*

Glyburide *(Diabeta)*

Glibenclamide (Euglucon)

Acarbose *(Glucobay)*

Repaglinide *(Gluconorm)*

Linagliptin *(Trajenta/Jentadueto)**

Saxagliptin *(Onglyza)**

Sitagliptin *(Januvia/Janumet)**

Pioglitazone *(Actos)*

Rosiglitazone *(Avandia/Avandamet)*

Dapagliflozin *(Forxiga)**

Empagliflozin *(Jardiance)**

Canagliflozin *(Invokana)**

Linagliptin/Metformin HCL *(Jentadueto)**

Sitagliptin / Metformin HCL *(Janumet)**

Komboglyza*

**Other Blood Pressure Medications**

Clonidone *(Catapres/Clonidine)*

Methyldopa (Aldomet)

Hydralazine (Apresoline)

Minoxidil *(Loniten)*

Doxazosin *(Cardura)*

Prazosin *(Prazo)*

Terazosin *(Hytrin)*

**Anticoagulants**

Warfarin *(Coumadin)*

Rivaroxaban *(Xarelto)**

Dabigatran *(Pradaxa)**

Apixaban *(Eliquis)**

Dalteparin *(Fragmin)*

Tinzaparin *(Innohep)*

Enoxaparin *(Lovenox)*

Heparin *(Heparin Leo)*

Nadroparin *(Fraxiparine)*

Fondaparinux *(Arixtra)*

Danaparoid *(Orgaran)*

**Insulin**

Insulin Aspart (Novorapid)

Insulin Detemir (Levemir)

Insulin Glargine *(Lantus)*

Insulin Glulisine *(Apidra)*

Insulin R *(Novolin/Humulin)*

Insulin Lispro *(Humalog)*

Insulin Humulin 30/70

Insulin Humulin N

Insulin Humulin R

Insulin Novolin NPH

Insulin Novolin Toronto

Insulin Novolin Mix (30/70, 40/60, 50/50)

**Smoking Cessation Aids**

Varenicline *(Champix)*

*your physician must complete additional paperwork for this medication to be covered by Blue Cross. If you are paying the full cost of this medication, please check with your pharmacist or physician.

***For medications which have been around for a long time, many equivalent versions of the same medicine are available (known as generic medications). For these medications, the brand name medication, although it is has been shown to be equivalent to the generic medications, often costs several fold more than generic medications, and Blue Cross does not pay for the more expensive equivalent version. People can choose to pay to receive the more expensive equivalent medication. In the ACCESS study, copayments are eliminated only for the less expensive generic medications.*
