# Supplementary material for: Cost analysis and efficacy of recruitment strategies used in a large pragmatic community-based clinical trial targeting low-income seniors: a comparative descriptive analysis
Source: Trials. 2019 Oct 7;20:577. doi: 10.1186/s13063-019-3652-5 (PMC6781395; doi:10.1186/s13063-019-3652-5)
Supplement: Supplementary file 2 — Telephone Baseline Questionnaire. (DOCX 66 kb) [file 13063_2019_3652_MOESM2_ESM.docx]

Additional file 2: Telephone Baseline Questionnaire

| **1** | **TZONE** |
| --- | --- |
| Single | |
| Min = 1 Max = 1 L = 2 | |
| 13/04/2017 8:35:00 PM | |
| **This question computes automatically the time zones corresponding with the imported phone numbers, more precisely with the area codes of these phone numbers. This only applies in North America, as this is the only continent which has the same phone number format everywhere within (3 first digits for the area codes, NNX for the three following digits and the local phone numbers with the last four digits).** | |
| ==> /+11 > 0 | |
|  | |
| ==> Roster | |
| Position: 1 | |
| \| Choices \| \| \| \| \| \| \| --- \| --- \| --- \| --- \| --- \| --- \| \| Newfoundland \| 17 \|  \|  \|  \|  \| \| Atlantic \| 19 \|  \|  \|  \|  \| \| Eastern \| 13 \|  \|  \|  \|  \| \| Central \| 12 \|  \|  \|  \|  \| \| Saskatchewan \| 10 \|  \|  \|  \|  \| \| Mountains \| 07 \|  \|  \|  \|  \| \| Pacific \| 05 \|  \|  \|  \|  \| | |

| **2** | **LANG** |
| --- | --- |
| Single | |
| Min = 1 Max = 1 L = 2 | |
| 13/04/2017 8:35:00 PM | |
| ==> /+11 > 0 | |
|  | |
| ==> Roster | |
| Position: 3 | |

| **3** | **PIN_CODE** |
| --- | --- |
| Single | |
| Min = 1 Max = 1 L = 4 | |
| 13/04/2017 8:35:00 PM | |
| PIN CODE | |
| ==> /+11 > 0 | |
|  | |
| ==> Roster | |
| Position: 5 | |

| **4** | **SOURCE** |
| --- | --- |
| Single | |
| Min = 1 Max = 1 L = 2 | |
| 13/04/2017 8:35:00 PM | |
| **How was the contact obtained** | |
|  | |
| ==> Roster | |
| Position: 9 | |
| \| Choices \| \| \| \| \| \| \| --- \| --- \| --- \| --- \| --- \| --- \| \| Voice message \| 01 \|  \|  \|  \|  \| \| List \| 02 \|  \|  \|  \|  \| | |

| **5** | **SDATE** |
| --- | --- |
| Single | |
| Min = 1 Max = 1 L = 8 | |
| 13/04/2017 8:35:00 PM | |
| **Start Date in YYYYMMDD Format. Date when the voicemail or list was been received.** | |
| $C 20160101 20200101 | |
| ==> Roster | |
| Position: 11 | |

| **6** | **PHONE** |
| --- | --- |
| Single | |
| Min = 1 Max = 1 L = 20 | |
| 13/04/2017 8:35:00 PM | |
| **Primary Phone Number PHONE in (XXX)XXX-XXXX format.** | |
| (999)999-9999 | |
| ==> Roster | |
| Position: 19 | |

| **7** | **FNAME** |
| --- | --- |
| Single | |
| Min = 0 Max = 1 L = 50 | |
| 13/04/2017 8:35:00 PM | |
| **First Name** | |
|  | |
| ==> Roster | |
| Position: 39 | |

| **8** | **LNAME** |
| --- | --- |
| Single | |
| Min = 0 Max = 1 L = 50 | |
| 13/04/2017 8:35:00 PM | |
| **Last Name** | |
|  | |
| ==> Roster | |
| Position: 89 | |

| **9** | **INTRO** |
| --- | --- |
| Single | |
| Min = 1 Max = 1 L = 2 | |
| 13/04/2017 8:35:00 PM | |
| **ACCESS Phone: <PHONE> Check Area Code: <PHONE> Long Distance Code: 1167117-9-1 CB Message: <NAME>** | |
| ==> INT97$A > 12 | |
|  | |
| ==> Roster | |
| Position: 139 | |
| \| Choices \| \| \| \| \| \| \| --- \| --- \| --- \| --- \| --- \| --- \| \| Successful Connection \| SU \| D \| ==> /INT01 \|  \|  \| \| Busy \| BU \|  \| ==> /END \|  \|  \| \| No Answer \| NA \|  \| ==> /END \|  \|  \| \| Answering Machine \| AM \|  \| ==> /END \|  \|  \| \| Fast Busy (redial and try long distance) \| FB \|  \| ==> /END \|  \|  \| \| Fax Machine (redial after 60 seconds) \| FX \|  \| ==> /END \|  \|  \| \| NIS or Disconnected Number (redial) \| DC \|  \| ==> /END \|  \|  \| \| Callback Failure (re-code by end of shift) \| CF \|  \| ==> /NAME \|  \|  \| | |

| **10** | **INT01** |
| --- | --- |
| Single | |
| Min = 1 Max = 1 L = 2 | |
| 13/04/2017 8:35:00 PM | |
| **Hello, this is $I from Primary Data Support at Alberta Health Services calling regarding the enrollment in the ACCESS study. May I please speak to <FNAME> <LNAME>? FOR PARTIAL INTERVIEW: ONLY RESUME INTERVIEW IF NAME IS GIVEN. May I speak with . .? Would now be a good time to complete the survey? If you recall you were answering questions about the ACCESS study. CB Message: <NAME> IF NO NAME WAS LEFT ON VOICEMAIL: `May I please speak to the person that recently called a toll-free number regarding the ACCESS study?´** | |
|  | |
| ==> Roster | |
| Position: 141 | |
| \| Choices \| \| \| \| \| \| \| --- \| --- \| --- \| --- \| --- \| --- \| \| Yes, continue interview \| 01 \|  \| ==> /FIRST_T \|  \|  \| \| No, do not continue interview \| 02 \|  \| ==> /INT \|  \|  \| \| Resume partial interview (Only if NAME in callback) \| RI \|  \| ==> /LASTQ \|  \|  \| | |

| **11** | **FIRST_T** |
| --- | --- |
| Single | |
| Min = 1 Max = 1 L = 2 | |
| 13/04/2017 8:35:00 PM | |
| **Is this the first time you were calling the 1-844 number (the number where you left a voicemail):** | |
| ==> +1SOURCE = 02 | |
|  | |
| ==> Roster | |
| Position: 143 | |
| \| Choices \| \| \| \| \| \| \| --- \| --- \| --- \| --- \| --- \| --- \| \| Yes \| 01 \|  \|  \|  \|  \| \| No \| 02 \|  \|  \|  \|  \| | |

| **12** | **COMPLETE** |
| --- | --- |
| Single | |
| Min = 1 Max = 1 L = 2 | |
| 13/04/2017 8:35:00 PM | |
| **Have you completed the entire ACCESS telephone survey before? YES: Stop the survey and code as DS. Advise to call Study Coordinator number to provide additional information. NO: Proceed as applicable: continue with survey (e.g. person has a Senior Blue Cross now), or stop the survey and code as D1 if want just general information.** | |
|  | |
| ==> Roster | |
| Position: 145 | |
| \| Choices \| \| \| \| \| \| \| --- \| --- \| --- \| --- \| --- \| --- \| \| Yes \| 01 \|  \| ==> /INT \|  \|  \| \| No \| 02 \|  \|  \|  \|  \| | |

| **13** | **INPERSON** |
| --- | --- |
| Single | |
| Min = 1 Max = 1 L = 2 | |
| 13/04/2017 8:35:00 PM | |
| **Are you the person interested in the study or you are calling on behalf of another person?** | |
|  | |
| ==> Roster | |
| Position: 147 | |
| \| Choices \| \| \| \| \| \| \| --- \| --- \| --- \| --- \| --- \| --- \| \| Person interested to participate \| 01 \|  \|  \|  \|  \| \| Calling on behalf of other \| 02 \|  \|  \|  \|  \| | |

| **14** | **FNAME2** |
| --- | --- |
| Single | |
| Min = 1 Max = 1 L = 30 | |
| 13/04/2017 8:35:00 PM | |
| **IF TALKING TO SENIOR PARTICIPANT: What is your first name? IF TALKING TO PROXY: What is the first name of the senior participant?** | |
|  | |
| ==> Roster | |
| Position: 149 | |

| **15** | **LNAME2** |
| --- | --- |
| Single | |
| Min = 1 Max = 1 L = 30 | |
| 13/04/2017 8:35:00 PM | |
| **IF TALKING TO SENIOR PARTICIPANT: What is your last name? IF TALKING TO PROXY: What is the last name of the senior participant?** | |
| ==> INT021 = 1 | |
|  | |
| ==> Roster | |
| Position: 179 | |

| **16** | **PFNAME** |
| --- | --- |
| Single | |
| Min = 1 Max = 1 L = 30 | |
| 13/04/2017 8:35:00 PM | |
| **Could you please confirm your first name Enter stated name (of proxy respondent)** | |
| ==> INT02INPERSON = 01 | |
|  | |
| ==> Roster | |
| Position: 209 | |

| **17** | **PLNAME** |
| --- | --- |
| Single | |
| Min = 1 Max = 1 L = 30 | |
| 13/04/2017 8:35:00 PM | |
| **Could you please confirm your last name Enter stated last name (of proxy respondent)** | |
|  | |
| ==> Roster | |
| Position: 239 | |

| **18** | **WHYP** |
| --- | --- |
| Multiple , Open | |
| Min = 1 Max = 4 L = 2 | |
| 13/04/2017 8:35:00 PM | |
| **Why is <FNAME2> <LNAME2> not able to answer the questions himself/herself, is it because he/she is: READ OPTIONS AND CHECK ALL THAT APPLY:** | |
|  | |
| ==> Roster | |
| Position: 269 | |
| \| Choices \| \| \| \| \| \| \| --- \| --- \| --- \| --- \| --- \| --- \| \| Hard of hearing \| 01 \|  \|  \|  \|  \| \| Does not speak English \| 02 \|  \|  \|  \|  \| \| Is disabled (could not provide oral or written responses) \| 03 \|  \|  \|  \|  \| \| Has dementia or cognitively impaired \| 04 \| X \| ==> /INT \|  \|  \| \| Other, specify; \| 05 \| O \|  \|  \|  \| | |

| **19** | **PROXY_YN** |
| --- | --- |
| Single | |
| Min = 1 Max = 1 L = 2 | |
| 13/04/2017 8:35:00 PM | |
| **Could the senior person you are calling on behalf of, independently complete further consent form and surveys about his or her health condition and medications? All this material will be in English. Would you say... READ OPTIONS** | |
|  | |
| ==> Roster | |
| Position: 277 | |
| \| Choices \| \| \| \| \| \| \| --- \| --- \| --- \| --- \| --- \| --- \| \| Yes, senior can complete materials independently \| 01 \|  \|  \|  \|  \| \| No, senior can not complete materials independently \| 02 \|  \|  \|  \|  \| | |

| **20** | **ASSIST** |
| --- | --- |
| Single | |
| Min = 1 Max = 1 L = 2 | |
| 13/04/2017 8:35:00 PM | |
| **Please be aware that if the person you are calling on behalf of is eligible for this study, you are agreeing to assist him or her in the study enrollment process. This includes: - Reviewing and signing a consent form that we will mail to <FNAME2> <LNAME2> - Assisting <FNAME2> <LNAME2> in filling out a short survey about his or her medical history. Are you able to commit to assist <FNAME2> <LNAME2> with this? READ OPTIONS** | |
| ==> AWAREPROXY_YN = 01 | |
|  | |
| ==> Roster | |
| Position: 279 | |
| \| Choices \| \| \| \| \| \| \| --- \| --- \| --- \| --- \| --- \| --- \| \| Yes \| 01 \|  \|  \|  \|  \| \| No \| 02 \|  \| ==> /INT11 \|  \|  \| | |

| **21** | **AWARE** |
| --- | --- |
| Single | |
| Min = 1 Max = 1 L = 2 | |
| 13/04/2017 8:35:00 PM | |
| **Is the participant aware that you are calling on their behalf? If NO (Not aware), READ: Please note that in order to fully complete this survey on behalf of the senior participant, you will require some of his or her personal information such as personal health number, medication list and home address. NO: Advise that they need to inform participant. Book a call-back with proxy)** | |
|  | |
| ==> Roster | |
| Position: 281 | |
| \| Choices \| \| \| \| \| \| \| --- \| --- \| --- \| --- \| --- \| --- \| \| Yes \| 01 \|  \|  \|  \|  \| \| No \| 02 \|  \| ==> /CB \|  \|  \| | |

| **22** | **PERSAWARE** |
| --- | --- |
| Single | |
| Min = 1 Max = 1 L = 2 | |
| 13/04/2017 8:35:00 PM | |
| **Later in the script we will ask you to provide some personal information about participant. Is the participant aware you are providing his/her personal information to us? If asked what kind of personal information, READ: Personal information being personal health number, medication list and home address. NO: Advise that they need to inform participant. Book a call-back with proxy** | |
|  | |
| ==> Roster | |
| Position: 283 | |
| \| Choices \| \| \| \| \| \| \| --- \| --- \| --- \| --- \| --- \| --- \| \| Yes \| 01 \|  \|  \|  \|  \| \| No \| 02 \|  \| ==> /CB \|  \|  \| | |

| **23** | **OTHERL** |
| --- | --- |
| Single , Open | |
| Min = 1 Max = 1 L = 2 | |
| 13/04/2017 8:35:00 PM | |
| **What is <FNAME2> <LNAME2> native language? IF ASKED WHY ARE YOU ASKING THIS: We need this information so that we can prepare short printed information about the study for the patient in specified language.** | |
| Else ==> PERSONWHYP = 02 | |
|  | |
| ==> Roster | |
| Position: 285 | |
| \| Choices \| \| \| \| \| \| \| --- \| --- \| --- \| --- \| --- \| --- \| \| French \| 01 \|  \|  \|  \|  \| \| Punjabi \| 02 \|  \|  \|  \|  \| \| Urdu \| 03 \|  \|  \|  \|  \| \| German \| 04 \|  \|  \|  \|  \| \| Mandarin \| 05 \|  \|  \|  \|  \| \| Cantonese \| 06 \|  \|  \|  \|  \| \| Tagalog \| 07 \|  \|  \|  \|  \| \| Vietnamese \| 08 \|  \|  \|  \|  \| \| Korean \| 09 \|  \|  \|  \|  \| \| Spanish \| 10 \|  \|  \|  \|  \| \| Arabic \| 11 \|  \|  \|  \|  \| \| Other, specify \| 12 \| O \|  \|  \|  \| | |

| **24** | **PERSON** |
| --- | --- |
| Single | |
| Min = 1 Max = 1 L = 2 | |
| 13/04/2017 8:35:00 PM | |
| **Please answer the following questions as if you are the senior person interested in the Study.** | |
|  | |
| ==> Roster | |
| Position: 287 | |
| \| Choices \| \| \| \| \| \| \| --- \| --- \| --- \| --- \| --- \| --- \| \| OK \| 01 \| D \|  \|  \|  \| | |

| **25** | **INT02** |
| --- | --- |
| Single | |
| Min = 1 Max = 1 L = 2 | |
| 13/04/2017 8:35:00 PM | |
| **Thank you for your interest in this important study. The ACCESS study is being conducted by researchers from the University of Calgary who are interested in looking at ways to improve outcomes for people with chronic conditions. This study has been reviewed and approved by the University of Calgary's Health Research Ethics board. We are working with the ACCESS Study team to enroll participants. In order to assess eligibility to participate in this study, I have a couple of short questions to ask you. This should only take about 10 minutes. Is this a good time for you to talk?** | |
|  | |
| ==> Roster | |
| Position: 289 | |
| \| Choices \| \| \| \| \| \| \| --- \| --- \| --- \| --- \| --- \| --- \| \| DO NOT READ: Continue, good time to talk \| 01 \| D \|  \|  \|  \| \| DO NOT READ: Not a good time to talk \| 02 \|  \| ==> /INT \|  \|  \| | |

| **26** | **INT03** |
| --- | --- |
| Single | |
| Min = 1 Max = 1 L = 2 | |
| 13/04/2017 8:35:00 PM | |
| **This survey is confidential. AHS collects health information in accordance with Section 20 of the Health Information Act for the purpose of providing health services or any other purpose authorized under section 27 of the Act. If you have any questions or concerns regarding the collection, use or disclosure of your information for this survey, please let me know and I can provide you with the contact information for the study coordinator. If yes, person would like contact information, read: ACCESS study coordinator number is 1-844-310-0585. Any information you provide is voluntary and you can stop the interview at any time. This call may be monitored for Quality Control purposes. May I continue?** | |
|  | |
| ==> Roster | |
| Position: 291 | |
| \| Choices \| \| \| \| \| \| \| --- \| --- \| --- \| --- \| --- \| --- \| \| Yes, continue interview \| 01 \|  \|  \|  \|  \| \| No, do not continue interview \| 02 \|  \| ==> /INT \|  \|  \| | |

| **27** | **WHERE** |
| --- | --- |
| Single , Open | |
| Min = 1 Max = 1 L = 2 | |
| 13/04/2017 8:35:00 PM | |
| **How did you hear about the ACCESS study? DO NOT READ OPTIONS** | |
|  | |
| ==> Roster | |
| Position: 293 | |
| \| Choices \| \| \| \| \| \| \| --- \| --- \| --- \| --- \| --- \| --- \| \| Poster in your pharmacy \| 01 \|  \|  \|  \|  \| \| Pharmacist handed you a brochure \| 02 \|  \|  \|  \|  \| \| Poster in doctor office \| 03 \|  \|  \|  \|  \| \| Received brochure at your doctor’s office \| 04 \|  \|  \|  \|  \| \| Community Newsletter / Newspaper \| 05 \|  \|  \|  \|  \| \| Friend or Family member \| 06 \|  \|  \|  \|  \| \| Other, specify \| 07 \| O \|  \|  \|  \| \| Community Senior’s newsletter \| 08 \|  \|  \|  \|  \| \| Media (print or TV advertisement) \| 09 \|  \|  \|  \|  \| \| Radio \| 10 \|  \|  \|  \|  \| \| Social Media \| 11 \|  \|  \|  \|  \| \| DO NOT READ: Don't know \| 99 \| X \|  \|  \|  \| \| DO NOT READ: Refused \| 98 \| X \|  \|  \|  \| | |

| **28** | **DOB** |
| --- | --- |
| Single | |
| Min = 1 Max = 1 L = 8 | |
| 13/04/2017 8:35:00 PM | |
| **What is your birth date? Birth date of senior participant.** | |
| $D /split | |
| ==> Roster | |
| Position: 295 | |

| **29** | **TODAY** |
| --- | --- |
| Single | |
| Min = 1 Max = 1 L = 8 | |
| 13/04/2017 8:35:00 PM | |
| **Computed question to return today's date** | |
| ==> *$D | |
| $D | |
| ==> Roster | |
| Position: 303 | |

| **30** | **AGE** |
| --- | --- |
| Single | |
| Min = 1 Max = 1 L = 3 | |
| 13/04/2017 8:35:00 PM | |
| ==> *YEA(TODAY) - YEA(DOB) - (V01(MON(DOB) >MON(TODAY))) - (V01(MON(DOB) == MON(TODAY)) AND DAY(DOB) > DAY (TODAY))) | |
|  | |
| ==> Roster | |
| Position: 311 | |

| **31** | **DISPLAY** |
| --- | --- |
| Single | |
| Min = 1 Max = 1 L = 1 | |
| 13/04/2017 8:35:00 PM | |
| **DOB : <DOB> Today : <TODAY> Age : <AGE>** | |
| ==> INT11Else ==> BLUECAGE | |
|  | |
| ==> Roster | |
| Position: 314 | |
| \| Choices \| \| \| \| \| \| \| --- \| --- \| --- \| --- \| --- \| --- \| \| OK \| 1 \|  \|  \|  \|  \| | |

| **32** | **BLUEC** |
| --- | --- |
| Single | |
| Min = 1 Max = 1 L = 2 | |
| 13/04/2017 8:35:00 PM | |
| **Do you have Alberta Blue Cross Coverage for Seniors coverage that pays for most of your Medications? If don't know, ask them to check, and if they confirm, recode as 'Yes'. Keep as 'Don't know if they cannot confirm. If don´t have Alberta blue cross Coverage code as DQ, and advise to call ACCESS coordinator that can assist with obtaining this coverage. Also advise to call back again the 1-844 number and leave the new message once the Blue Cross for Seniors is obtained).** | |
|  | |
| ==> Roster | |
| Position: 315 | |
| \| Choices \| \| \| \| \| \| \| --- \| --- \| --- \| --- \| --- \| --- \| \| Yes \| 01 \|  \|  \|  \|  \| \| No \| 02 \|  \| ==> /INT11 \|  \|  \| \| I have applied but haven’t received the card \| 03 \|  \| ==> /INT11 \|  \|  \| \| DO NOT READ: Don't know \| 99 \|  \| ==> /INT11 \|  \|  \| \| DO NOT READ: Refused \| 98 \|  \|  \|  \|  \| | |

| **33** | **OTHERIN** |
| --- | --- |
| Single | |
| Min = 1 Max = 1 L = 2 | |
| 13/04/2017 8:35:00 PM | |
| **Do you have any other health insurance that pays some or all of your prescription medication expenses? If 'yes' Ask the respondent to identify the name of this other insurance. If they have Enhanced Blue Cross Insurance (Seniors Plus) - code as ``No´´ (continue interview). If they have Seniors Benefit, Senior Financial Assistance or Special Needs Assistance or any other insurance - code as `Yes´ (discontinue interview).** | |
|  | |
| ==> Roster | |
| Position: 317 | |
| \| Choices \| \| \| \| \| \| \| --- \| --- \| --- \| --- \| --- \| --- \| \| Yes \| 01 \|  \| ==> /INT11 \|  \|  \| \| No \| 02 \|  \|  \|  \|  \| \| DO NOT READ: Don't know \| 99 \|  \| ==> /INT11 \|  \|  \| \| DO NOT READ: Refused \| 98 \|  \| ==> /INT11 \|  \|  \| | |

| **34** | **MED_TAKN** |
| --- | --- |
| Single | |
| Min = 1 Max = 1 L = 2 | |
| 13/04/2017 8:35:00 PM | |
| **Does a nurse or other professional caregiver provide you with your medications each day?** | |
|  | |
| ==> Roster | |
| Position: 319 | |
| \| Choices \| \| \| \| \| \| \| --- \| --- \| --- \| --- \| --- \| --- \| \| Yes \| 01 \|  \| ==> /INT11 \|  \|  \| \| No \| 02 \|  \|  \|  \|  \| \| DO NOT READ: Don't know \| 99 \|  \|  \|  \|  \| \| DO NOT READ: Refused \| 98 \|  \|  \|  \|  \| | |

| **35** | **INCOME** |
| --- | --- |
| Single | |
| Min = 1 Max = 1 L = 2 | |
| 13/04/2017 8:35:00 PM | |
| **Which of the following categories represents your annual household income (before taxes or deductions)? Household income being... READ OPTIONS AND STOP WHEN RESPONSE IS REACHED** | |
|  | |
| ==> Roster | |
| Position: 321 | |
| \| Choices \| \| \| \| \| \| \| --- \| --- \| --- \| --- \| --- \| --- \| \| Less than $15,000 \| 01 \|  \|  \|  \|  \| \| $15,000 - $29,999 \| 02 \|  \|  \|  \|  \| \| $30,000 - $50,000 \| 03 \|  \|  \|  \|  \| \| Over 50,000 \| 04 \|  \| ==> /INT11 \|  \|  \| \| DO NOT READ: Don't know \| 99 \|  \| ==> /INT11 \|  \|  \| \| DO NOT READ: Refused \| 98 \|  \| ==> /INT11 \|  \|  \| | |

| **36** | **HEART** |
| --- | --- |
| Single | |
| Min = 1 Max = 1 L = 2 | |
| 13/04/2017 8:35:00 PM | |
| **Have you ever been told by a doctor that you have coronary heart disease (for example Angina, heart attack, blocked arteries, stents or bypass surgery)?** | |
|  | |
| ==> Roster | |
| Position: 323 | |
| \| Choices \| \| \| \| \| \| \| --- \| --- \| --- \| --- \| --- \| --- \| \| Yes \| 01 \|  \|  \|  \|  \| \| No \| 02 \|  \|  \|  \|  \| \| DO NOT READ: Don't know \| 99 \|  \|  \|  \|  \| \| DO NOT READ: Refused \| 98 \|  \|  \|  \|  \| | |

| **37** | **STROKE** |
| --- | --- |
| Single | |
| Min = 1 Max = 1 L = 2 | |
| 13/04/2017 8:35:00 PM | |
| **Have you ever been told by a doctor that you have had a stroke? NOTE: TIA (Transient Ischemic Attack) or a mini-stroke, this does not qualify as a stroke. Code as NO.** | |
|  | |
| ==> Roster | |
| Position: 325 | |
| \| Choices \| \| \| \| \| \| \| --- \| --- \| --- \| --- \| --- \| --- \| \| Yes \| 01 \|  \|  \|  \|  \| \| No \| 02 \|  \|  \|  \|  \| \| DO NOT READ: Don't know \| 99 \|  \|  \|  \|  \| \| DO NOT READ: Refused \| 98 \|  \|  \|  \|  \| | |

| **38** | **KIDNEY** |
| --- | --- |
| Single | |
| Min = 1 Max = 1 L = 2 | |
| 13/04/2017 8:35:00 PM | |
| **Have you ever been told by a doctor that you have chronic kidney disease? IF ASKED TO CLARIFY: Generally patients with chronic kidney disease don´t experience any symptoms and it is detected through blood tests, so in general, the only way to know if you have kidney disease is if your physician has told you that you have weak kidneys or kidney disease or failure, or if they have talked to you about the possibility of needing to go on dialysis at some point in the future.** | |
|  | |
| ==> Roster | |
| Position: 327 | |
| \| Choices \| \| \| \| \| \| \| --- \| --- \| --- \| --- \| --- \| --- \| \| Yes \| 01 \|  \|  \|  \|  \| \| No \| 02 \|  \|  \|  \|  \| \| DO NOT READ: Don't know \| 99 \|  \|  \|  \|  \| \| DO NOT READ: Refused \| 98 \|  \|  \|  \|  \| | |

| **39** | **HEARTF** |
| --- | --- |
| Single | |
| Min = 1 Max = 1 L = 2 | |
| 13/04/2017 8:35:00 PM | |
| **Have you ever been told by a doctor that you have heart failure, a weakened heart, or had water in your lungs? IF ASKED TP CLARIFY: Heart failure is a term used to describe a weakened heart muscle - which often causes shortness of breath or leg swelling. One´s heart can become weak for a variety of reasons, including prior heart attack, blood clots in the lungs, heart rhythm abnormalities, viruses and various medications and toxins. A doctor may describe heart failure as `having water in lungs´ or `fluid that backs up into the legs´. Patients with heart failure have usually had an abnormal heart ultrasound (echocardiogram) and may be treated with salt restriction and water pills/diuretics like Lasix/furosemide.** | |
|  | |
| ==> Roster | |
| Position: 329 | |
| \| Choices \| \| \| \| \| \| \| --- \| --- \| --- \| --- \| --- \| --- \| \| Yes \| 01 \|  \|  \|  \|  \| \| No \| 02 \|  \|  \|  \|  \| \| DO NOT READ: Don't know \| 99 \|  \|  \|  \|  \| \| DO NOT READ: Refused \| 98 \|  \|  \|  \|  \| | |

| **40** | **DIABET** |
| --- | --- |
| Single | |
| Min = 1 Max = 1 L = 2 | |
| 13/04/2017 8:35:00 PM | |
| **Have you ever been told by a doctor that you have diabetes? IF REQUIRED This means at any point in time (past or present)** | |
|  | |
| ==> Roster | |
| Position: 331 | |
| \| Choices \| \| \| \| \| \| \| --- \| --- \| --- \| --- \| --- \| --- \| \| Yes \| 01 \|  \|  \|  \|  \| \| No \| 02 \|  \|  \|  \|  \| \| DO NOT READ: Don't know \| 99 \|  \|  \|  \|  \| \| DO NOT READ: Refused \| 98 \|  \|  \|  \|  \| | |

| **41** | **CHOLES** |
| --- | --- |
| Single | |
| Min = 1 Max = 1 L = 2 | |
| 13/04/2017 8:35:00 PM | |
| **Have you ever been told by a doctor that you have high cholesterol? IF REQUIRED This means at any point in time (past or present)** | |
|  | |
| ==> Roster | |
| Position: 333 | |
| \| Choices \| \| \| \| \| \| \| --- \| --- \| --- \| --- \| --- \| --- \| \| Yes \| 01 \|  \|  \|  \|  \| \| No \| 02 \|  \|  \|  \|  \| \| DO NOT READ: Don't know \| 99 \|  \|  \|  \|  \| \| DO NOT READ: Refused \| 98 \|  \|  \|  \|  \| | |

| **42** | **BLOODP** |
| --- | --- |
| Single | |
| Min = 1 Max = 1 L = 2 | |
| 13/04/2017 8:35:00 PM | |
| **Have you ever been told by a doctor that you have high blood pressure? IF REQUIRED This means at any point in time (past or present) If self monitored high blood pressure, record as Yes** | |
|  | |
| ==> Roster | |
| Position: 335 | |
| \| Choices \| \| \| \| \| \| \| --- \| --- \| --- \| --- \| --- \| --- \| \| Yes \| 01 \|  \|  \|  \|  \| \| No \| 02 \|  \|  \|  \|  \| \| DO NOT READ: Don't know \| 99 \|  \|  \|  \|  \| \| DO NOT READ: Refused \| 98 \|  \|  \|  \|  \| | |

| **43** | **SMOKE** |
| --- | --- |
| Single | |
| Min = 1 Max = 1 L = 2 | |
| 13/04/2017 8:35:00 PM | |
| **Do you currently smoke tobacco products (cigarettes, pipes, cigars) OPTIONAL READ: E-cigarettes do not qualify as smoking** | |
|  | |
| ==> Roster | |
| Position: 337 | |
| \| Choices \| \| \| \| \| \| \| --- \| --- \| --- \| --- \| --- \| --- \| \| Yes \| 01 \|  \|  \|  \|  \| \| No \| 02 \|  \|  \|  \|  \| \| DO NOT READ: Refused \| 98 \|  \|  \|  \|  \| | |

| **44** | **SMOKE20** |
| --- | --- |
| Single | |
| Min = 1 Max = 1 L = 2 | |
| 13/04/2017 8:35:00 PM | |
| **For the majority of the time that you have been smoking, did you smoke half pack or more per day?** | |
| ==> CONTINUENOT SMOKE = 01 | |
|  | |
| ==> Roster | |
| Position: 339 | |
| \| Choices \| \| \| \| \| \| \| --- \| --- \| --- \| --- \| --- \| --- \| \| Yes \| 01 \|  \|  \|  \|  \| \| No \| 02 \|  \|  \|  \|  \| \| DO NOT READ: Don't know \| 99 \|  \|  \|  \|  \| \| DO NOT READ: Refused \| 98 \|  \|  \|  \|  \| | |

| **45** | **CONTINUE** |
| --- | --- |
| Single | |
| Min = 1 Max = 1 L = 2 | |
| 13/04/2017 8:35:00 PM | |
| **Based on your responses, you are eligible for this study. As you know, the purpose of this study is to look at ways to improve health for people with chronic health conditions like the ones you have. In this three year study, we are testing the impact of two interventions to which you would be randomly assigned: - receiving medications for diabetes, high blood pressure, or heart disease free of charge through your Blue Cross Drug plan (in other words without copayment) and - receiving a comprehensive patient education program tailored to you based on the information you provided to us at the start of the study. There is a 50% chance that you will receive selected medications free, and a 50% chance that you will receive personalized education. There is no cost for you to participate in this study. Are you interested in hearing more about this study?** | |
| Else ==> INT11(V01(HEART=01)+V01(STROKE=01)+V01(KIDNEY=01)+V01(HEARTF=01))>0 OR (V01(DIABET=01)+V01(CHOLES=01)+V01(BLOODP=01)+V01(SMOKE20=01))>1 | |
|  | |
| ==> Roster | |
| Position: 341 | |
| \| Choices \| \| \| \| \| \| \| --- \| --- \| --- \| --- \| --- \| --- \| \| Yes, continue \| 01 \|  \|  \|  \|  \| \| No, do not continue \| 02 \|  \| ==> /INT \|  \|  \| | |

| **46** | **CONSENT** |
| --- | --- |
| Single | |
| Min = 1 Max = 1 L = 2 | |
| 13/04/2017 8:35:00 PM | |
| **The ACCESS Study Team will send you more information about the study by mail or by email depending on your preference. This information will include a consent form that explains the study in detail and a study questionnaire. You will not be required to attend any meetings or travel for the purposes of this study. If you are selected to receive medications without copayments, there is nothing else you will need to do. ACCESS team will coordinate everything directly with the Alberta Blue Cross. We need to ask you a few questions over the phone before proceeding, including your contact information, preferred method of contact, and one question about your current medications. The information that you provide will only be used to allow them to contact you, and ensure you get the correct version of the questionnaire that they send you. We will never share your personal information with anyone. Are you willing to provide this information.** | |
|  | |
| ==> Roster | |
| Position: 343 | |
| \| Choices \| \| \| \| \| \| \| --- \| --- \| --- \| --- \| --- \| --- \| \| Yes, continue \| 01 \|  \|  \|  \|  \| \| No, do not continue \| 02 \|  \| ==> /INT \|  \|  \| | |

| **47** | **ADDRES_1** |
| --- | --- |
| Single | |
| Min = 1 Max = 1 L = 50 | |
| 13/04/2017 8:35:00 PM | |
| **What is your house number and street name (or PO BOX)** | |
| ==> TELNOINPERSON = 01 AND OTHERL > 01 | |
|  | |
| ==> Roster | |
| Position: 345 | |

| **48** | **ADDRES_2** |
| --- | --- |
| Single | |
| Min = 0 Max = 1 L = 50 | |
| 13/04/2017 8:35:00 PM | |
| **Municipality** | |
|  | |
| ==> Roster | |
| Position: 395 | |

| **49** | **ADDRES_3** |
| --- | --- |
| Single | |
| Min = 0 Max = 1 L = 50 | |
| 13/04/2017 8:35:00 PM | |
| **Province** | |
|  | |
| ==> Roster | |
| Position: 445 | |

| **50** | **ADDRES_4** |
| --- | --- |
| Single | |
| Min = 0 Max = 1 L = 10 | |
| 13/04/2017 8:35:00 PM | |
| **Postal Code** | |
| A9A9A9 | |
| ==> Roster | |
| Position: 495 | |

| **51** | **ADDRES_5** |
| --- | --- |
| Single | |
| Min = 0 Max = 1 L = 50 | |
| 13/04/2017 8:35:00 PM | |
| **Postal Box number and station (if applicable)** | |
|  | |
| ==> Roster | |
| Position: 505 | |

| **52** | **TELNO** |
| --- | --- |
| Single | |
| Min = 1 Max = 1 L = 30 | |
| 13/04/2017 8:35:00 PM | |
| **What is the most convenient telephone number if they need to contact you regarding the study related matters? Enter stated number. Format (XXX)XXX-XXXX Note this should be the phone number of senior patient** | |
| ==> TELNO1WHYP = 02,01 | |
| (999)999-9999 | |
| ==> Roster | |
| Position: 555 | |

| **53** | **TELNOTP** |
| --- | --- |
| Single | |
| Min = 1 Max = 1 L = 2 | |
| 13/04/2017 8:35:00 PM | |
| **Please specify the type of this telephone number. Is this your.... READ OPTIONS** | |
|  | |
| ==> Roster | |
| Position: 585 | |
| \| Choices \| \| \| \| \| \| \| --- \| --- \| --- \| --- \| --- \| --- \| \| Home number \| 01 \|  \|  \|  \|  \| \| Work number \| 02 \|  \|  \|  \|  \| \| Cell number \| 03 \|  \|  \|  \|  \| \| DO NOT READ: Refused \| 98 \|  \|  \|  \|  \| | |

| **54** | **TELNO1** |
| --- | --- |
| Single | |
| Min = 1 Max = 1 L = 30 | |
| 13/04/2017 8:35:00 PM | |
| **What is your telephone number? Enter stated number of the proxy respondent. Format (XXX)XXX-XXXX** | |
| ==> TELNO2INPERSON = 01 | |
| (999)999-9999 | |
| ==> Roster | |
| Position: 587 | |

| **55** | **TELNO2** |
| --- | --- |
| Single | |
| Min = 0 Max = 1 L = 30 | |
| 13/04/2017 8:35:00 PM | |
| **If your phone service was to change, or we are unable to reach you, can you please provide an alternate contact number of a family member or a friend? Enter stated number. Format (XXX)XXX-XXXX We do not need to record their name. This number has to be different from the primary contact number.** | |
| ==> AHSNO_YNINPERSON = 02 | |
| (999)999-9999 | |
| ==> Roster | |
| Position: 617 | |

| **56** | **AHSNO_YN** |
| --- | --- |
| Single | |
| Min = 1 Max = 1 L = 2 | |
| 13/04/2017 8:35:00 PM | |
| **Now I need to ask you for your Alberta Health Care Number. This number will be used to change your Alberta Blue Cross insurance, if you are selected to receive your preventative medications for free, and to link to your health records. Please make sure to read the 9-digit number slowly. It can be found on your Alberta health care card. What is your Alberta Health Care number?** | |
|  | |
| ==> Roster | |
| Position: 647 | |
| \| Choices \| \| \| \| \| \| \| --- \| --- \| --- \| --- \| --- \| --- \| \| DO NOT READ: Yes, willing provide PHN \| 01 \| D \| ==> /AHSNO \|  \|  \| \| DO NOT READ: No, refused to provide \| 02 \|  \| ==> /INT11 \|  \|  \| | |

| **57** | **AHSNO** |
| --- | --- |
| Single | |
| Min = 1 Max = 1 L = 23 | |
| 13/04/2017 8:35:00 PM | |
| **Enter stated number. Enter number in "XXXXX-XXXX" format** | |
| 99999-9999 | |
| ==> Roster | |
| Position: 649 | |

| **58** | **AHSNO_CONF** |
| --- | --- |
| Single | |
| Min = 1 Max = 1 L = 2 | |
| 13/04/2017 8:35:00 PM | |
| **To ensure we have the correct number, could you please confirm that your Alberta Health Care Number is: <AHSNO>** | |
|  | |
| ==> Roster | |
| Position: 672 | |
| \| Choices \| \| \| \| \| \| \| --- \| --- \| --- \| --- \| --- \| --- \| \| Yes \| 01 \| X \|  \|  \|  \| \| No \| 02 \| X \| ==> /AHSNO \|  \|  \| | |

| **59** | **MEDIC** |
| --- | --- |
| Multiple | |
| Min = 1 Max = 52 L = 4 | |
| 13/04/2017 8:35:00 PM | |
| Medications | |
| **Now a question about medications. We are going to ask specific questions about your medications. Could you please get your prescription medications or current medication list prior to beginning to answer these questions. Please read your medication list to me slowly - only the names of the drugs; doses and times of day are not necessary. CHECK MATCHED MEDICATIONS ONLY. IF NAME IS NOT ON THE LIST, DISREGARD, DO NOT RECORD. IF ASKED: WHY ARE YOUR ASKING / WHY DO YOU NEED TO KNOW: 'This information is needed so that the Research Team can send you appropriate study material based on the medications you are taking.** | |
|  | |
| ==> Roster | |
| Position: 674 | |
| \| Choices \| \| \| \| \| \| \| --- \| --- \| --- \| --- \| --- \| --- \| \| Accupril \| A9B \|  \|  \|  \|  \| \| Altace \| A8B \|  \|  \|  \|  \| \| Atacand \| B1B \|  \|  \|  \|  \| \| Atorvastatin \| C1A \|  \|  \|  \|  \| \| Avalide \| B3C \|  \|  \|  \|  \| \| Avapro \| B3B \|  \|  \|  \|  \| \| Benazepril \| 41A \|  \|  \|  \|  \| \| Candesartan \| B1A \|  \|  \|  \|  \| \| Capoten \| A5B \|  \|  \|  \|  \| \| Captopril \| A5A \|  \|  \|  \|  \| \| Cilazapril \| A2A \|  \|  \|  \|  \| \| Coversyl \| A4B \|  \|  \|  \|  \| \| Cozaar \| B4B \|  \|  \|  \|  \| \| Crestor \| C2B \|  \|  \|  \|  \| \| Diovan \| B6B \|  \|  \|  \|  \| \| Enalapril \| A3A \|  \|  \|  \|  \| \| Eprosartan \| B2A \|  \|  \|  \|  \| \| Fluvastatin \| C5A \|  \|  \|  \|  \| \| Fosinopril \| A6A \|  \|  \|  \|  \| \| Hyzaar \| B4C \|  \|  \|  \|  \| \| Inhibace \| A2B \|  \|  \|  \|  \| \| Irbesartan \| B3A \|  \|  \|  \|  \| \| Leschol \| C5B \|  \|  \|  \|  \| \| Lipitor \| C1B \|  \|  \|  \|  \| \| Lisinopril \| A7A \|  \|  \|  \|  \| \| Losartan \| B4A \|  \|  \|  \|  \| \| Lotensin \| 41B \|  \|  \|  \|  \| \| Lovastatin \| C6A \|  \|  \|  \|  \| \| Mavik \| A10B \|  \|  \|  \|  \| \| Mevacor \| C6B \|  \|  \|  \|  \| \| Micardis \| B5B \|  \|  \|  \|  \| \| Monopril \| A6B \|  \|  \|  \|  \| \| Olmesartan \| B7A \|  \|  \|  \|  \| \| Olmetec \| B7B \|  \|  \|  \|  \| \| Perindopril \| A4A \|  \|  \|  \|  \| \| Prevachol \| C4B \|  \|  \|  \|  \| \| Pravastatin \| C4A \|  \|  \|  \|  \| \| Prinivil \| A7C \|  \|  \|  \|  \| \| Quinapril \| A9A \|  \|  \|  \|  \| \| Ramipril \| A8A \|  \|  \|  \|  \| \| Rosuvastatin \| C2A \|  \|  \|  \|  \| \| Simvastatin \| C3A \|  \|  \|  \|  \| \| Telmisartan \| B5A \|  \|  \|  \|  \| \| Teveten \| B2B \|  \|  \|  \|  \| \| Trandolapril \| A10A \|  \|  \|  \|  \| \| Twynsta \| B5C \|  \|  \|  \|  \| \| Valsartan \| B6A \|  \|  \|  \|  \| \| Vaseretic \| A3C \|  \|  \|  \|  \| \| Vasotec \| A3B \|  \|  \|  \|  \| \| Zestoretic \| A7D \|  \|  \|  \|  \| \| Zestril \| A7B \|  \|  \|  \|  \| \| Zocor \| C3B \|  \|  \|  \|  \| \| DO NOT READ: Refused / Don't know \| 0053 \|  \|  \|  \|  \| \| DO NOT READ: Not Applicable / Not on list \| 0054 \|  \|  \|  \|  \| | |

| **60** | **CONTACT** |
| --- | --- |
| Multiple | |
| Min = 1 Max = 2 L = 2 | |
| 13/04/2017 8:35:00 PM | |
| **Finally, we want to know what is the best way to contact you to send you the consent form, questionnaires and educational materials relevant for this study. There are 2 ways to participate: via email communication, or through regular (post) mail. If email communication is selected, your enrolment process may be accelerated due to eliminated postage waiting times. However, if you are not comfortable navigating emails and logging onto websites, you may choose to select regular mail. You are free to change your preferences throughout the study by calling our study coordinator. How would you like to receive the material for the study? READ OPTIONS: CHECK ALL THAT APPLY** | |
| ==> INT99INPERSON = 02 | |
|  | |
| ==> Roster | |
| Position: 882 | |
| \| Choices \| \| \| \| \| \| \| --- \| --- \| --- \| --- \| --- \| --- \| \| Email and Internet website \| 01 \|  \|  \|  \|  \| \| Regular mail \| 02 \|  \|  \|  \|  \| | |

| **61** | **EMAIL** |
| --- | --- |
| Single | |
| Min = 1 Max = 1 L = 60 | |
| 13/04/2017 8:35:00 PM | |
| **What is your email address MAKE SURE THIS IS THE EMAIL OF SENIOR PARTICIPANT Enter stated email. Read and confirm spelling.** | |
| Else ==> CELLYNCONTACT = 01 | |
| $@ | |
| ==> Roster | |
| Position: 886 | |

| **62** | **PIN** |
| --- | --- |
| Single | |
| Min = 1 Max = 1 L = 2 | |
| 13/04/2017 8:35:00 PM | |
| **Over the next few days, you will receive an email inviting you to visit a secure website where you can receive further information about the study. To access the website, you will need a four digit number, I will provide this four digit number to you now. The ACCESS team will also provide you this number in the mail. If necessary wait for person to obtain pen and paper FOUR DIGIT PIN: <PIN_CODE>** | |
|  | |
| ==> Roster | |
| Position: 946 | |
| \| Choices \| \| \| \| \| \| \| --- \| --- \| --- \| --- \| --- \| --- \| \| DO NOT READ: 4 digit number was recorded \| 01 \|  \|  \|  \|  \| \| DO NOT READ: Refused to record 4 digit number \| 02 \|  \|  \|  \|  \| | |

| **63** | **CELLYN** |
| --- | --- |
| Single | |
| Min = 1 Max = 1 L = 2 | |
| 13/04/2017 8:35:00 PM | |
| **If you are selected to receive educational materials to help you manage your chronic disease, this will be done by email or mail, according to your preference. Would you also like to receive these materials via SMS or Text messaging on your cellular phone? SMS is text messaging used on cellular phones.** | |
|  | |
| ==> Roster | |
| Position: 948 | |
| \| Choices \| \| \| \| \| \| \| --- \| --- \| --- \| --- \| --- \| --- \| \| Yes \| 01 \|  \|  \|  \|  \| \| No \| 02 \|  \| ==> /INT99 \|  \|  \| \| DO NOT READ: Not applicable, no cell phone \| 03 \|  \| ==> /INT99 \|  \|  \| | |

| **64** | **CELNO** |
| --- | --- |
| Single | |
| Min = 0 Max = 1 L = 30 | |
| 13/04/2017 8:35:00 PM | |
| **Could you provide your cell number? Enter Cell number of senior participantstated number (XXX)XXX-XXXX format Cell number of senior participant If cell phone number already provided continue to next question** | |
| Else ==> INT99CELLYN = 01 | |
| (999)999-9999 | |
| ==> Roster | |
| Position: 950 | |

| **65** | **INT11** |
| --- | --- |
| Single | |
| Min = 1 Max = 1 L = 2 | |
| 13/04/2017 8:35:00 PM | |
| **IF NOT MEETING CRITERIA: Unfortunately you will not qualify for this study. At this time we are only collecting information from persons that fit into certain categories and your circumstances are different than those. Thank you very much for taking the time to speak with me. IF NO PHN NUMBER GIVEN: Unfortunately you will not qualify for this study. The study requires all participants to provide the Personal Health Care number in order to change the insurance for selected participants. Thank you very much for taking the time to speak with me. PROXY NOT COMMITING (CODE DP): I am sorry but we will not be able to continue this call as you indicated that you are not able to commit to assist <FNAME2> <LNAME2> during the course of this program. Please advise another family member or a friend that will be able to assist, to call our 1-844 944 8927 number and leave a message. Thank you very much for taking the time to speak with me.** | |
| ==> INT99CELLYN = 01,02 | |
|  | |
| ==> Roster | |
| Position: 980 | |
| \| Choices \| \| \| \| \| \| \| --- \| --- \| --- \| --- \| --- \| --- \| \| Disqualified \| DQ \|  \| ==> /END \|  \|  \| \| Disqualified Proxy \| DP \|  \| ==> /END \|  \|  \| | |

| **66** | **INT97** |
| --- | --- |
| Single | |
| Min = 1 Max = 1 L = 2 | |
| 13/04/2017 8:35:00 PM | |
| Call Count Exceeded | |
| **Record has been called more than 12 times.** | |
|  | |
| ==> Roster | |
| Position: 982 | |
| \| Choices \| \| \| \| \| \| \| --- \| --- \| --- \| --- \| --- \| --- \| \| Call Count Exceeded \| CE \| D \| ==> /END \|  \|  \| | |

| **67** | **INT99** |
| --- | --- |
| Single | |
| Min = 1 Max = 1 L = 2 | |
| 13/04/2017 8:35:00 PM | |
| **Over the next week, you will receive all necessary information about the study. Please note that in order to be officially enrolled in this study you will need to agree to the consent form and to complete the health related questionnaire they will send you. If you have any additional questions, you may call the Study Coordinator at: 1-844-310-0585 Those are all the questions I have to ask you for this survey. Thank you for your time. Have a nice evening / good day. Goodbye. Survey Completed, Duration : $T** | |
|  | |
| ==> Roster | |
| Position: 984 | |
| \| Choices \| \| \| \| \| \| \| --- \| --- \| --- \| --- \| --- \| --- \| \| Finish \| FN \| D \| ==> /END \|  \|  \| | |

| **68** | **INT** |
| --- | --- |
| Single | |
| Min = 1 Max = 1 L = 2 | |
| 13/04/2017 8:35:00 PM | |
| Refusal Screen | |
| **GENERAL EXIT I'm sorry to have bothered you, thank you for your time. Have a good day/evening. LANGUAGE/COMMUNICATION BARRIER: I'm sorry, but I will be unable to complete the survey with you at this time. Thank you for your interest. Have a good day/evening. IF ALREADY COMPLETED ACCESS: Exit Survey and Code DS IF REFUSE TO DO THE SURVEY (OR NOT INTERESTED) Code RE DECEASED: I am so sorry to hear about your loss. I'm sorry to have bothered you. Thank you for your time. Have a good day/evening. GENERAL EXIT I am sorry but cognitively impaired seniors are not eligible for the study, as they may not benefit from the educational materials that are planned for the study. Thank you for your time. Have a good day/evening** | |
|  | |
| ==> Roster | |
| Position: 986 | |
| \| Choices \| \| \| \| \| \| \| --- \| --- \| --- \| --- \| --- \| --- \| \| Refusal - General \| RE \|  \| ==> /END \|  \|  \| \| Language Barrier (ESL) \| LB \|  \| ==> /END \|  \|  \| \| Unable to Communicate (non-ESL) \| UH \|  \| ==> /END \|  \|  \| \| Wrong Number \| WN \|  \| ==> /END \|  \|  \| \| Hostile Interrupt (respondent ends survey, no callback) \| HI \|  \| ==> /END \|  \|  \| \| Partial Interview (book callback) \| PI \|  \| ==> /NAME \|  \|  \| \| Incomplete Interview (no callback) \| IC \|  \| ==> /END \|  \|  \| \| Callback General \| CG \|  \| ==> /NAME \|  \|  \| \| Callback Person (qualified) \| CP \|  \| ==> /NAME \|  \|  \| \| Deceased \| DD \|  \| ==> /END \|  \|  \| \| Disqualified - end survey \| DQ \|  \| ==> /END \|  \|  \| \| (INTRO) Successful Connection \| SU \| N \|  \|  \|  \| \| (INTRO) Busy \| BU \| N \|  \|  \|  \| \| (INTRO) Fast Busy (redial and try long distance) \| FB \| N \|  \|  \|  \| \| (INTRO) No Answer \| NA \| N \|  \|  \|  \| \| (INTRO) Answering Machine \| AM \| N \|  \|  \|  \| \| (INTRO) Fax Machine (redial fater 60 seconds) \| FX \| N \|  \|  \|  \| \| (INTRO) NIS or Disconnected Number (redial) \| DC \| N \|  \|  \|  \| \| (INTRO) Callback Failure \| CF \| N \|  \|  \|  \| \| (INT99) Finish \| FN \| N \|  \|  \|  \| \| (INT01) Yes, continue interview \| 01 \| N \|  \|  \|  \| \| (INT01) No, do not continue interview \| 02 \| N \|  \|  \|  \| \| Time out \| W0 \| N \|  \|  \|  \| \| More than one session attempted on the same case \| W1 \| N \|  \|  \|  \| \| Project inactivated while session in progress \| W2 \| N \|  \|  \|  \| \| Disconnected by supervisor \| W3 \| N \|  \|  \|  \| \| (INT01) Resume partial interview (Only if NAME in callback) \| RI \| N \|  \|  \|  \| \| Reset Case \| RS \| N \|  \|  \|  \| \| (INTRO) Business (redial/confirm) \| BZ \| N \|  \|  \|  \| \| (INT97) Call Count Exceeded \| CE \|  \| ==> /END \|  \|  \| \| Duplicate Survey \| DS \|  \| ==> /END \|  \|  \| \| Information Only DO NOT COMPLETE SURVEY \| D1 \|  \| ==> /END \|  \|  \| \| (INT11) Disqualified Proxy \| DP \| N \|  \|  \|  \| | |

| **69** | **F8** |
| --- | --- |
| Single , Open | |
| Min = 1 Max = 1 L = 2 | |
| 13/04/2017 8:35:00 PM | |
| General Notes | |
| **General Notes** | |
|  | |
| ==> Roster | |
| Position: 988 | |
| \| Choices \| \| \| \| \| \| \| --- \| --- \| --- \| --- \| --- \| --- \| \| Enter to continue \| 01 \| DO \|  \|  \|  \| | |

| **70** | **F9** |
| --- | --- |
| Single | |
| Min = 1 Max = 1 L = 2 | |
| 13/04/2017 8:35:00 PM | |
| Study Contact No | |
| **]If there are any further questions about the study, you can contact the ACCESS Study Coordinator at: 1-844-310-0585** | |
|  | |
| ==> Roster | |
| Position: 990 | |

| **71** | **F11** |
| --- | --- |
| Single | |
| Min = 1 Max = 1 L = 1 | |
| 13/04/2017 8:35:00 PM | |
| Call History | |
|  | |
| ==> Roster | |
| Position: 992 | |

| **72** | **F10** |
| --- | --- |
| Single , Open | |
| Min = 1 Max = 1 L = 2 | |
| 13/04/2017 8:35:00 PM | |
| Question specific notes | |
|  | |
| ==> Roster | |
| Position: 993 | |
| \| Choices \| \| \| \| \| \| \| --- \| --- \| --- \| --- \| --- \| --- \| \| Enter question specific notes \| 01 \| DO \|  \|  \|  \| | |

| **73** | **NAME** |
| --- | --- |
| Single | |
| Min = 1 Max = 1 L = 80 | |
| 13/04/2017 8:35:00 PM | |
| **Name or notes** | |
|  | |
| ==> Roster | |
| Position: 995 | |

| **74** | **CB** |
| --- | --- |
| Single | |
| Min = 1 Max = 1 L = 12 | |
| 13/04/2017 8:35:00 PM | |
| **CURRENT TIME $H CURRENT DATE $D When would you like me to call you back? Please note you do not have to call us back at our 1-844 number. We will call you back to continue with the survey. Thank you for your time, Goodbye.** | |
| ==> /END$A>20 | |
| $CHS/V | |
| ==> Roster | |
| Position: 1075 | |
