# Supplementary material for: Cost analysis and efficacy of recruitment strategies used in a large pragmatic community-based clinical trial targeting low-income seniors: a comparative descriptive analysis
Source: Trials. 2019 Oct 7;20:577. doi: 10.1186/s13063-019-3652-5 (PMC6781395; doi:10.1186/s13063-019-3652-5)
Supplement: Supplementary file 3 — Paper Baseline Survey. (DOCX 612 kb) [file 13063_2019_3652_MOESM3_ESM.docx]

## SOME INFORMATION ABOUT YOU

1. What is your gender?

Man

Woman

Other

1. What is your marital status?

Married/Common-Law

Single

Divorced/Separated

Widowed

1. What is the highest level of education you completed?

Less than high school

High school

Some post-secondary

Post-secondary diploma/certificate

Bachelor’s degree or higher

1. What is your current employment status?

Employed full-time

Employed part-time

Retired

Unemployed

Caregiver/homemaker

1. Your current or last occupation fits best in the following category: (choose one only)

Management occupations

Business, finance, and administration occupations

Natural and applied sciences and related occupations

Health occupations

Occupations in education, law and social, community and government services

Occupations in art, culture, recreation and sport

Sales and service occupations

Trades, transport, and equipment operators and related occupations

Natural resources, agriculture and related production occupations

Occupations in manufacturing and utilities

Other-specify: ______________________________________________

1. What type of home best describes your living situation?

I live independently in a house, condo or apartment

I live in an assisted living facility like a senior’s lodge or Group home

I live in a Nursing home or long-term care facility

Other _____________________

1. How many people live in your household? (including yourself)

1

2

3

4

More than 4

1. Where were you born?

Canada

United States

Latin America/Caribbean

Europe

South Asia (India, Pakistan, Bangladesh, Sri Lanka)

East Asia/Southeast Asia

Africa

Other

9. IF YOU WEREN’T BORN IN CANADA, how long have you lived in Canada?

Less than 1 year

1-2 years

3-5 years

5-10 years

More than 10 years

1. What is your first/native language?

English

French

Other: _________________

1. How would you describe your ethnicity/race?

Caucasian/White

Visible Minority

Aboriginal: First Nations/Inuit/Metis

## SOME INFORMATION ABOUT YOUR HEALTH AND HEALTH CARE

1. Do you have a regular medical doctor?

Yes

No

Don’t Know

1. In general, would you say your mental health is...?

Excellent

Very good

Good

Fair

Poor

1. Over the last two weeks, how often have you felt little interest or pleasure in doing things?

Nearly every day

More than half the days

Several days

Not at all

Don’t Know

1. Over the last two weeks, how often have you felt down, depressed, or hopeless?

Nearly every day

More than half the days

Several days

Not at all

Don’t Know

1. Have you been diagnosed with: (check all that apply)

Depression/Dysthymia

Anxiety

Fibromyalgia

Chronic pain

Chronic fatigue syndrome

1. What is your approximate weight? (report in **EITHER** pounds or kilograms)

_____________ Pounds **OR** ___________ Kilograms

1. What is your approximate height? (report in **EITHER** feet/inches or centimeters)

__________Feet, __________Inches **OR** _______________ Centimeters

**If you HAVE DIABETES, please answer questions 19 and 20, otherwise skip to question 21.**

1. **IF** you have Diabetes, what type of diabetes do you have?

Type 1 (onset in youth, immediately treated with insulin)

Type 2 (onset in adulthood, first treated with lifestyle and/or pills before insulin)

Unsure

1. How is your diabetes treated?

Diet and exercise only

Pills to lower blood sugar

Insulin therapy

Pills and insulin

**If you HAVE CHRONIC KIDNEY DISEASE, please answer question 21, otherwise skip to question 22**

1. **IF** you have Chronic Kidney Disease, how is it treated?

Medications only

Peritoneal dialysis

Hemodialysis

Kidney transplant

### **If you are NOT currently smoking, ANSWER THIS PAGE.**

**If you ARE currently smoking, skip to question #24 on the following page.**

1. Were you ever a smoker in the past?

Yes

No - **proceed to page 11**

1. **IF** you were previously a smoker, when did you quit smoking?

Within the last 6 months

More than 6 months ago

**Answer this page ONLY if you are CURRENTLY A SMOKER**

**If you are currently a NONSMOKER, skip to question 26 on the following page.**

1. In the last year, how many times have you quit smoking for at least 24 hours?

0

Once

Twice

More than twice

1. Are you thinking seriously of quitting smoking?

Yes, within the next 30 days

Yes, within the next 6 months

No

## Now we have some questions about your medications.

## ALL respondents should answer this section

26. How many different prescription medications are you taking on a regular or ongoing basis? Please include all prescribed medications you are taking.

0

1-3

4-6

> 6

1. Do you ever forget to take your medicine?

Yes

No

1. Are you careless at times about taking your medicine?

Yes

No

1. When you feel better do you sometimes stop taking your medicine?

Yes

No

1. Sometimes if you feel worse when you take the medicine, do you stop taking it?

Yes

No

1. In the past month, did you take aspirin or any other ASA (acetylsalicylic acid) medication every day or every second day? (Not including Tylenol or Advil)

Yes

No

**Section 1:**

The following medications are called Statins:

Atorvastatin (*Lipitor*) Rosuvastatin (*Crestor*) Simvastatin (*Zocor*) Pravastatin (*Pravachol*) Fluvastatin (*Lescol*) Lovastatin (*Mevacor*)

If you are **NOT** currently taking a statin (or are unsure), **GO TO QUESTION 32** on **page 14**.

If you **ARE** currently taking a statin, **SKIP TO QUESTION 34** on **page 15**

1. Were you ever prescribed one of these medications IN THE PAST, but no longer take them?

Yes

No (skip to **SECTION 2** on page 16)

Don’t know (skip to **SECTION 2** on page 16)

33. **If YES**, why did you stop taking your statin? (Check all that apply)

Forgot to refill medication Side effects caused by medication

Cost Medication not working

Ran out of medication Condition controlled without medication

Feels ok without medication/doesn’t need medication

Do not know how to take medication properly

Don’t know

Other-specify: ______________________________________________________________

If you **ARE** currently taking a Statin, answer this page.

If you are **NOT** currently taking a statin, skip to **SECTION 2** on the following page.

1. People often have difficulty taking their pills for one reason or another. In the past week, how many days did you **take** your statin medication?

Not once

1-2 days

3-4 days

5-6 days

All 7 days **(skip to SECTION 2** on page 16**).**

1. Why did you miss some doses of your statin? (check all that apply)

Forget to take medication Side effects caused by medication

Cost Medication not working

Ran out of medication Condition controlled without medication

Feels ok without medication/doesn’t need medication

Do not know how to take medication properly

Don’t know

Other-specify:

### **Section 2:**

The following medications are called ACE Inhibitors or Angiotensin Receptor Blockers:

| **Ace-Inhibitors** | **Angiotensin receptor blockers** |
| --- | --- |
| Benazepril (Lotensin) | Candesartan (Atacand) |
| Cilazepril (Inhibace/ Inhibace Plus) | Eprosartan (Teveten) |
| Enalapril (Vasotec/ Vaseretic) | Irbesartan (Avapro/ Avalide) |
| Perindopril (Coversyl/ Coversyl Plus) | Losartan (Cozar/ Hyzaar) |
| Captopril (Capoten) | Telmisartan (Micardis/ Twynsta) |
| Fosinopril (Monopril) | Valsartan (Diovan) |
| Lisinopril (Zestril/ Prinivil/ Zestoretic) | Olmesartan (Olmetec/ Olmetec Plus) |
| Ramipril (Altace) |  |
| Quinapril (Accupril/ Accuretic) |  |
| Trandolapril (Mavik) |  |

If you are **NOT** currently taking an ACE Inhibitor or Angiotensin Receptor Blocker, **GO TO QUESTION #36** on the following page (**page 17**)

If you **ARE** currently taking an ACE inhibitor or Angiotensin Receptor Blocker, **SKIP TO QUESTION #38** on **page 18**

1. Have you ever been prescribed one of these medications in the past, but no longer take them?

Yes

No **(skip to question 38** on page 18)

Don’t know **(skip to question 38** on page 18)

37. **If YES**, why did you stop taking that medication? (Check all that apply)

Forgot to refill medication Side effects caused by medication

Cost Medication not working

Ran out of medication Condition controlled without medication

Feels ok without medication/doesn’t need medication

Do not know how to take medication properly

Don’t know

Other-specify:

If you **ARE** currently taking an ACE Inhibitor, or Angiotensin Receptor Blocker medication, **answer this page.**

If you are **NOT** currently taking an ACE Inhibitor, or Angiotensin Receptor Blocker medication, **SKIP TO THE FOLLOWING PAGE.**

1. People often have difficulty taking their pills for one reason or another. In the past week, how many days did you **take** your prescribed ACE Inhibitor or Angiotensin Receptor Blocker?

Not once

1-2 days

3-4 days

5-6 days

All 7 days **(skip to question 40** on pg. 19**).**

1. Why did you miss some doses of this medication? (check all that apply)

Forget to take medication Side effects caused by medication

Cost Medication not working

Ran out of medication Condition controlled without medication

Feels ok without medication/doesn’t need medication

Do not know how to take medication properly

Don’t know

Other-specify:

Barriers to receiving Health Care

## ALL respondents should answer this section

1. In the past 12 months, did you ever experience any difficulties getting services from your family doctor or specialist for the health conditions that qualified you for this study?

(Heart Failure, Heart Disease, Stroke, Chronic Kidney Disease, Diabetes, High Cholesterol, High Blood Pressure, or a history of Smoking)

Yes

No (**skip to question #42** on the following page)

Don’t know (**skip to question #42** on the following page)

1. If YES, what type of difficulties did you experience? (Check all that apply)


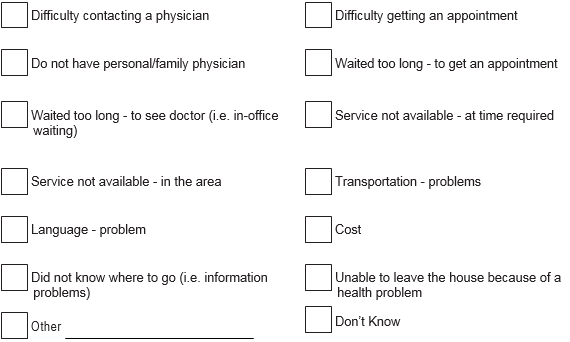


1. In the past 12 months, how often have you had difficulty paying for medications, services, or equipment you need to help manage your chronic condition?

(For instance, prescriptions, glucose testing strips for diabetes, or blood pressure cuffs etc.)

Always

Often

Sometimes

Rarely

Never (**skip to question #44** on the following page)

1. What type of services, equipment, or medication did you have a difficult time accessing due to cost? (Check all that apply)


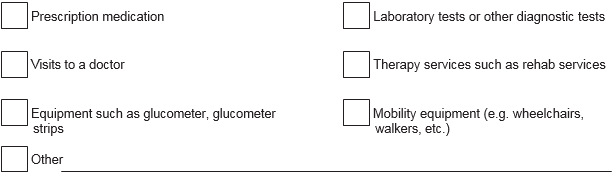


44. In the past 12 months, how often did you not get the necessary medications, services, or equipment you needed due to cost?

Always

Often

Sometimes

Rarely

Never

## SOME INFORMATION ABOUT HOW WELL YOU FUNCTION

1. Do you ever find you have problems in walking?

I’m unable to walk

I have severe problems in walking

I have moderate problems in walking

I have slight problems in walking

I have no problems in walking

1. Do you ever have problems washing or dressing yourself?

I am unable to wash or dress myself

I have severe problems washing or dressing myself

I have moderate problems washing or dressing myself

I have slight problems washing or dressing myself

I have no problems washing or dressing myself

1. Do you ever have problems doing your usual activities (e.g. work, study, housework, and family or leisure activities)?

I am unable to do my usual activities

I have severe problems doing my usual activities

I have moderate problems doing my usual activities

I have slight problems doing my usual activities

I have no problems doing my usual activities

1. Do you ever find yourself in pain or with discomfort?

I have extreme pain or discomfort

I have severe pain or discomfort

I have moderate pain or discomfort

I have slight pain or discomfort

I have no pain or discomfort

49. Do you ever feel anxious or depressed?

I am extremely anxious or depressed

I am severely anxious or depressed

I am moderately anxious or depressed

I am slightly anxious or depressed

I am not anxious or depressed

50. How confident are you filling out medical forms by yourself?

Very confident

Quite a bit confident

Somewhat confident

A little confident

Not at all confident

**How confident are you that you can take your prescription medications in the following scenarios:**

| **Questions** | **Not at all sure** | **A little sure** | **Fairly sure** | **Extremely sure** |
| --- | --- | --- | --- | --- |
| 51. When you are busy at home? |  |  |  |  |
| 52. When there is no one to remind you? |  |  |  |  |
| 53. When you worry about taking them for the rest of your life? |  |  |  |  |
| 54. When you do not have any symptoms? |  |  |  |  |
| 55. When you are with family members? |  |  |  |  |
| 56. When you are in a public place? |  |  |  |  |
| 57. When you are traveling? |  |  |  |  |
| 58. When you have other medication to take? |  |  |  |  |
| 59. When you feel well? |  |  |  |  |
| 60. How confident are you that you can make taking your medications part of your daily routine? |  |  |  |  |

We would like to ask you about your personal views about medicines prescribed for you. These are statements other people have made about their medicines. **Please indicate the extent to which you agree or disagree with them by ticking the appropriate box.**

**There are no right or wrong answers. We are interested in your personal views.**

| **Questions** | **Strongly disagree** | **Disagree** | **Neither agree nor disagree** | **Agree** | **Strongly Agree** |
| --- | --- | --- | --- | --- | --- |
| 61. I feel that my health at present depends on these medications |  |  |  |  |  |
| 62. I worry about having to take my medication |  |  |  |  |  |
| 63. I feel like life would be impossible without my medication |  |  |  |  |  |
| 64. Without my medication, I would be very ill |  |  |  |  |  |
| 65. I worry about the long term effects of my medication |  |  |  |  |  |
| 66. I feel that my medication is a mystery to me |  |  |  |  |  |
| 67. I think my health in the future will depend on my medication |  |  |  |  |  |
| 68. I feel that my medications disrupt my life |  |  |  |  |  |
| 69. I sometimes worry about becoming too dependent on my medication |  |  |  |  |  |
| 70. I think my medication protects me from becoming worse |  |  |  |  |  |

**Thank you for completing your baseline questionnaire for the ACCESS Study!**

**Please return this booklet, along with all pages of the completed**

**BLUE consent form (as per the included instructions)**

**Upon reception of your completed package, the ACCESS Team will randomize you into the study and provide you with any further necessary instructions.**

**If you have any questions, please contact our office at 1-844-310-0585**

If you would prefer to receive these surveys via email, and to participate in the study electronically through a secure website (instead of via regular mail), please provide your email address below or contact us at 1-844-310-0585

Email address: __________________________ (Please print clearly)
