# Supplementary material for: Cost analysis and efficacy of recruitment strategies used in a large pragmatic community-based clinical trial targeting low-income seniors: a comparative descriptive analysis
Source: Trials. 2019 Oct 7;20:577. doi: 10.1186/s13063-019-3652-5 (PMC6781395; doi:10.1186/s13063-019-3652-5)
Supplement: Supplementary file 4 — Summary of human resources costs. (DOCX 20 kb) [file 13063_2019_3652_MOESM4_ESM.docx]

| Recruitment Strategy | Research Coordinator  Cost^1^ (Hours worked)^2^ | Research Assistant #1  Cost (Hours worked)^3^ | Research Assistant #2  Cost (Hours worked) | Research Assistant #3  Cost (Hours worked) | Travel Costs | Additional Costs^4^ | Total Human Resources Cost |
| --- | --- | --- | --- | --- | --- | --- | --- |
| ***Health Care*** | ***0*** | ***$38,430 (1281)*** | ***$21,060 (702)*** | ***$9,720 (324)*** | ***$990*** | ***$18,110*** | ***$88,310*** |
| Pharmacies | 0 | $38,070 (1269) | $21,060 (702) | $9,720 (324) | $920 | $18,110 | $87,880 |
| Health professionals | 0 | $360 (12) | 0 | 0 | $70 | 0 | $430 |
| ***Paper Mail*** | ***$3,600 (60)*** | ***$300 (10)*** | ***0*** | ***0*** | ***0*** | ***$12,720*** | ***$16,620*** |
| Canada Post mail-out | 0 | $300 (10) | 0 | 0 | 0 | 0 | $300 |
| Coronary angiogram registry | $1,200 (20) | 0 | 0 | 0 | 0 | $12,720 | $13,920 |
| Contact after hospital discharge | $2,400 (40) | 0 | 0 | 0 | 0 | 0 | 2,400 |
| ***Media*** | ***$600 (10)*** | ***$2,820 (94)*** | ***$120 (4)*** | ***0*** | ***0*** | ***0*** | ***$3,540*** |
| *Paid Media* | *$600 (10)* | *$1,920 (64)* | *$120 (4)* | *0* | *0* | *0* | *$2,640* |
| Paid radio | 0 | $120 (4) | 0 | 0 | 0 | 0 | $120 |
| Facebook | 0 | $300 (10) | 0 | 0 | 0 | 0 | $300 |
| Hospital programming channel | 0 | $600 (20) | 0 | 0 | 0 | 0 | $600 |
| Transit advertising | 0 | 0 | $120 (4) | 0 | 0 | 0 | $120 |
| Print media | 0 | $900 (30) | 0 | 0 | 0 | 0 | $900 |
| *Unpaid Media* | 0 | *$900 (30)* | *0* | *0* | *0* | *0* | *$900* |
| ***Senior's Outreach*** | ***0*** | ***$2,220 (74)*** | ***$1,170 (39)*** | ***$540 (18)*** | ***$330*** | ***0*** | ***$4,260*** |
| Senior's Homes/Apartments | 0 | $2,130 (71) | $1,170 (39) | $540 (18) | $260 | 0 | $4,100 |
| Senior's Aid Resources | 0 | $90 (3) | 0 | 0 | $70 | 0 | $160 |
| ***Word of Mouth*** | ***0*** | ***$300 (10)*** | ***$300 (10)*** | ***$600 (20)*** | ***0*** | ***0*** | ***$1,200*** |
| **TOTAL COST (TOTAL HOURS)** | **$4,800 (80)** | **$44,070 (1469)** | **$22,650 (755)** | **$10,860 (362)** | **$1,320** | **$30,800** | **$113,930** |
|  |  |  |  |  |  |  |  |

Additional file 4. Summary of human resources cost broken down by the cost of the research coordinator’s and assistants time on each of the strategies. The number of total hours worked on that strategy is in brackets.

^1^All costs are in CAD.

^2^The hour worked represents the number of hours total the study member spent on the recruitment strategy. The research coordinator had an hourly wage of $60CAD/hour

^3^The research assistants had an hourly wage of $30CAD/hour.

^4^Additional costs represent human resources costs that were outside of the study team.
